# A Virtual Retina for Studying Population Coding

**DOI:** 10.1371/journal.pone.0053363

**Published:** 2013-01-14

**Authors:** Illya Bomash, Yasser Roudi, Sheila Nirenberg

**Affiliations:** Department of Physiology and Biophysics, Weill Medical College of Cornell University, New York, New York, United States of America; Instituto de Neurociencias de Alicante UMH-CSIC, Spain

## Abstract

At every level of the visual system – from retina to cortex – information is encoded in the activity of large populations of cells. The populations are not uniform, but contain many different types of cells, each with its own sensitivities to visual stimuli. Understanding the roles of the cell types and how they work together to form collective representations has been a long-standing goal. This goal, though, has been difficult to advance, and, to a large extent, the reason is data limitation. Large numbers of stimulus/response relationships need to be explored, and obtaining enough data to examine even a fraction of them requires a great deal of experiments and animals. Here we describe a tool for addressing this, specifically, at the level of the retina. The tool is a data-driven model of retinal input/output relationships that is effective on a broad range of stimuli – essentially, a virtual retina. The results show that it is highly reliable: (1) the model cells carry the same amount of information as their real cell counterparts, (2) the quality of the information is the same – that is, the posterior stimulus distributions produced by the model cells closely match those of their real cell counterparts, and (3) the model cells are able to make very reliable predictions about the functions of the different retinal output cell types, as measured using Bayesian decoding (electrophysiology) and optomotor performance (behavior). In sum, we present a new tool for studying population coding and test it experimentally. It provides a way to rapidly probe the actions of different cell classes and develop testable predictions. The overall aim is to build constrained theories about population coding and keep the number of experiments and animals to a minimum.

## Introduction

A fundamental goal in neuroscience is understanding population coding - that is, how information from the outside world is represented in the activity of populations of neurons [1]–[7]. For example, at every level of the visual system, information is arrayed across large populations of neurons. The populations are not homogeneous, but contain many different cell types, each having its own visual response properties [8]–[14]. Understanding the roles of the different cell types and how they work together to collectively encode visual scenes has been a long-standing problem.

One of the reasons this problem has been difficult to address is that the space of possible stimuli that needs to be explored is exceedingly large. For example, it is well known that there are retinal ganglion cells that respond preferentially to light onset and offset (referred to as ON cells and OFF cells, respectively). Numerous studies, however, have shown that these cells also have other properties, such as sensitivities to spatial patterns, motion, direction of motion, speed, noise, etc., leading to new ideas about what contributions these cells make to the overall visual representation [15]–[21]. Probing these sensitivities, or even a fraction of them, across all cell types, would require a great deal of experiments and an uncomfortably large number of animals.

Here we describe a tool for addressing this, specifically, at the level of the retina, and we vet it experimentally. Briefly, we recorded the responses of hundreds of retinal output cells (ganglion cells), modeled their input/output relationships, and constructed a virtual retina. It allows us to probe the system with many stimuli and generate hypotheses for how the different cell classes contribute to the overall visual representation.

To model the input/output relationships, we used a linear-nonlinear (LN) model structure. LN models have been applied to other problems, such as studying the role of noise correlations [22]. Here we show that they can serve another valuable function as well: studying the contributions of different cell classes to the representation of visual scenes. In addition, the models described here differ from other LN models in that they are effective for a broad range of stimuli, including those with complex statistics, such as spatiotemporally-varying natural scenes (see *Methods*).

The strength of this approach depends on how reliable its predictions are. We tested this three ways, and the results show that (1) the model cells carry the same amount of information as their real cell counterparts, (2) the quality of the information is the same, that is, the posterior stimulus distributions produced by the model cells closely match those of the real cells, and (3) the model cells are able to make very reliable predictions about the functions of the different ganglion cell classes, as measured using Bayesian decoding of electrophysiological data and behavioral performance on an optomotor task.

In sum, a major obstacle in studying high-dimensional problems, such as population coding with complex, heterogeneous populations, is that a great deal of exploratory research is needed. Exploratory research in live experiments is very slow and requires large numbers of animals. If one has a reliable model for the population, this problem can be greatly ameliorated. Here we describe such a model and then test it with both electrophysiological and behavioral assays. The results also provide new data about population coding, exemplifying the utility of the approach; specifically, the data support the growing notion that the ON and OFF retinal pathways are not simply mirror images of each other, but in fact have distinct signaling properties, particularly pertaining to the representation of motion information.

## Results

### Building the Data Set

We recorded the responses of several hundred mouse retinal ganglion cells (492 cells) to a broad range of stimuli, including binary white noise (checkerboards), natural scenes, and drifting sine wave gratings. We then constructed, for each cell, a model of its stimulus/response relationships using the binary white noise and a subset of the natural scenes, and then tested the models using the gratings and the remaining natural scene stimuli. Thus, the models were tested on both out-of-sample stimuli (natural scenes not used to construct the models) and stimuli with different spatial and temporal statistics (drifting gratings of different spatial and temporal frequencies).

Briefly, stimulus/response relationships were modeled using linear-nonlinear cascade models of the form,
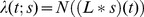
(1)


In this equation, the firing rate, 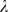, at time t, is produced by application of a linear filter, *L,* followed by a nonlinearity, *N*. For a general review of linear-nonlinear cascade models for retina and other systems, see refs. [23], [24]. For the functional forms and the parameter values of *L* and *N* that allow the models to capture stimulus/response relations over a broad range of stimuli, see refs. [25], [26].

### Assessing the Effectiveness of the Approach

To assess the effectiveness of the approach, we put it through a series of tests that measured both the *amount* of information carried by the model cells and the *quality* of the information carried by the model cells.

For the first, we used Shannon information: we measured the amount of information carried by each model cell and compared it to the amount of information carried by its corresponding real cell. For the second - for measuring the quality of the information - we used posterior stimulus distributions: we decoded each response produced by each model cell (using Bayesian decoding) and obtained the distribution of stimuli that resulted. We then compared this distribution to the distribution produced by decoding each response of the real cell. This allowed us to measure the extent to which the model cells’ responses mapped to the same distribution of stimuli as the real cells' responses, and, therefore, whether the model cells convey the same kind of information as the real cells. Finally, we also performed a bottom-line assessment: we used the model cells to make predictions about the functions of different ganglion cell classes, and then tested the predictions using Bayesian decoding (electrophysiology) and optomotor performance (behavior).

**Figure 1:**
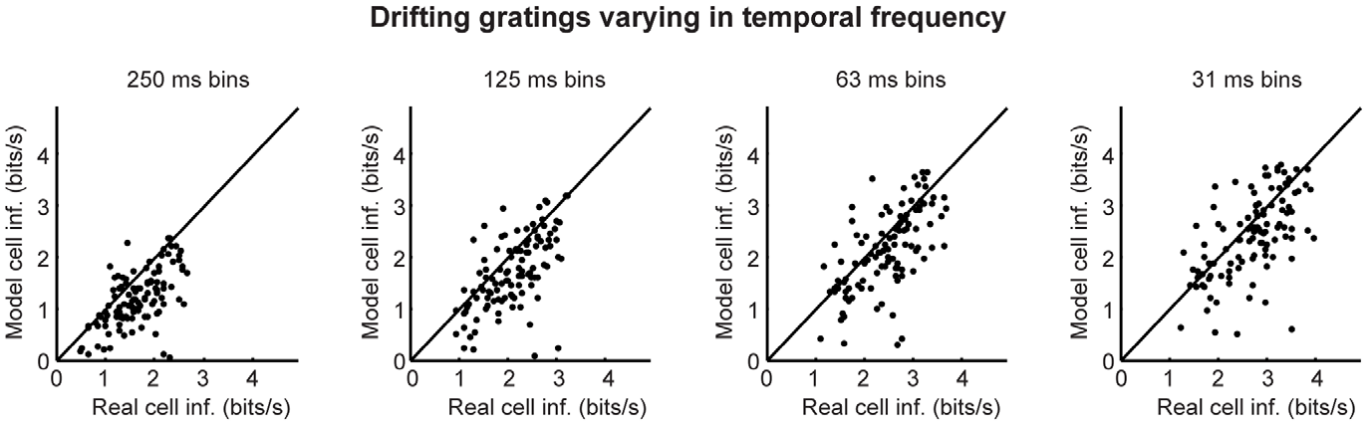
The amount of information carried by the model cells closely matched that of their real cell counterparts, when the stimulus set consisted of drifting gratings that varied in temporal frequency. The mutual information between each model cell’s responses and the stimuli was calculated and plotted against the mutual information between its corresponding real cell’s responses and the stimuli. Bin sizes ranged from 250 ms to 31 ms; *n* = 109 cells; stimulus entropy was 4.9 bits (30 one-second movie snippets). Note that there is scatter both above and below the line because of data limitation.

**Figure 2:**
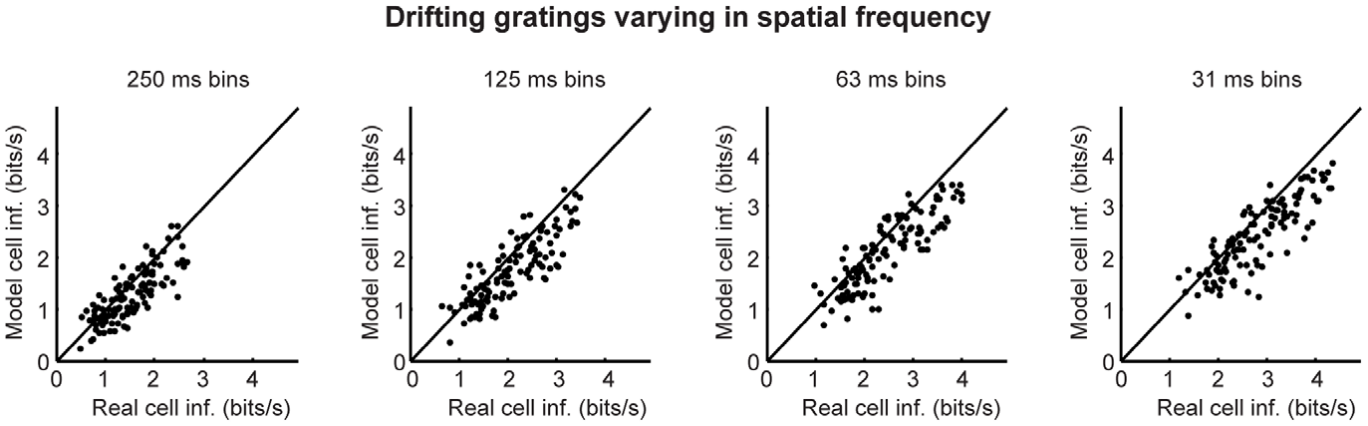
The amount of information carried by the model cells closely matched that of their real cell counterparts, when the stimulus set consisted of drifting gratings that varied in spatial frequency. As in Fig. 1, the mutual information between each model cell’s responses and the stimuli was calculated and plotted against the mutual information between its corresponding real cell’s responses and the stimuli. Bin sizes ranged from 250 ms to 31 ms; *n* = 120 cells; stimulus entropy was 4.9 bits (30 one-second movie snippets). Note that there is scatter both above and below the line because of data limitation.

**Figure 3:**
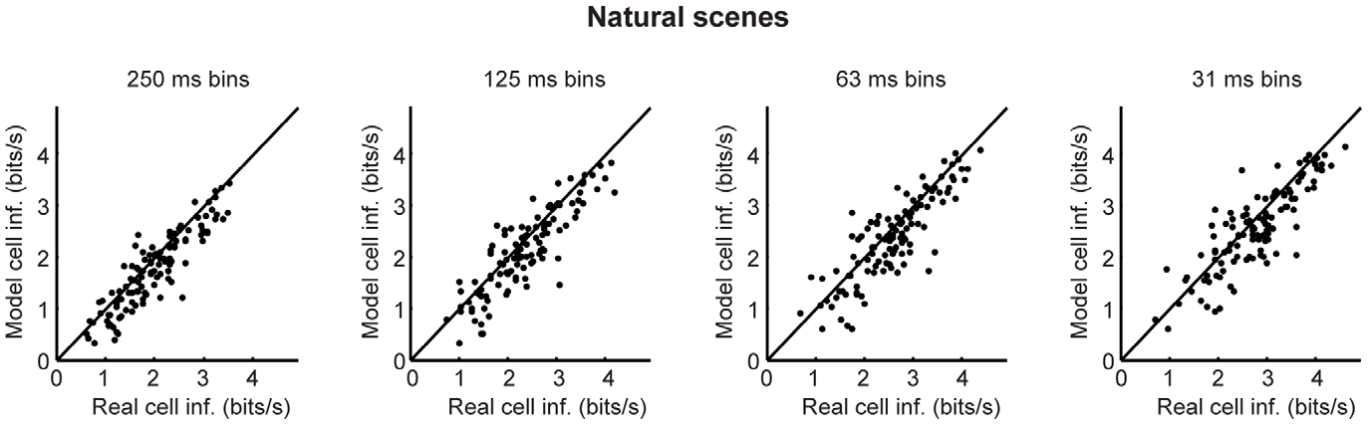
The amount of information carried by the model cells closely matched that of their real cell counterparts, when the stimulus set consisted of natural scene movies. As in Figs. 1 and 2, the mutual information between each model cell’s responses and the stimuli was calculated and plotted against the mutual information between its corresponding real cell’s responses and the stimuli. Bin sizes ranged from 250 ms to 31 ms; *n* = 113 cells; stimulus entropy was 4.9 bits (30 one-second movie snippets). Note that there is scatter both above and below the line because of data limitation.

Figs. 1, 2, 3 show the results of the Shannon information analysis. For this, we used three types of stimuli: drifting sine wave gratings that varied in temporal frequency, drifting sine wave gratings that varied in spatial frequency, and a set of natural scene movies. (As mentioned above and given in detail in *Methods,* the stimuli used to test the models were different from those used to build the models: the models were built using binary white noise and a different set of natural scenes.) For each model cell, we calculated the mutual information between its responses and the stimuli and compared it to the same for its real cell counterpart. As shown in the figures, the model cells carried nearly all the information carried by the real cells: using the finest bins, which give the most conservative values (i.e, the highest information for real cells), 91% of the information carried by the real cells was captured by the models: the mean over all cells and all stimuli was 91.1%; the median was 90.1. The robustness of the results was then checked by performing the calculations multiple times, each time increasing the temporal resolution used to characterize the responses by a factor of two until the processing time needed for the calculation became prohibitive.

**Figure 4:**
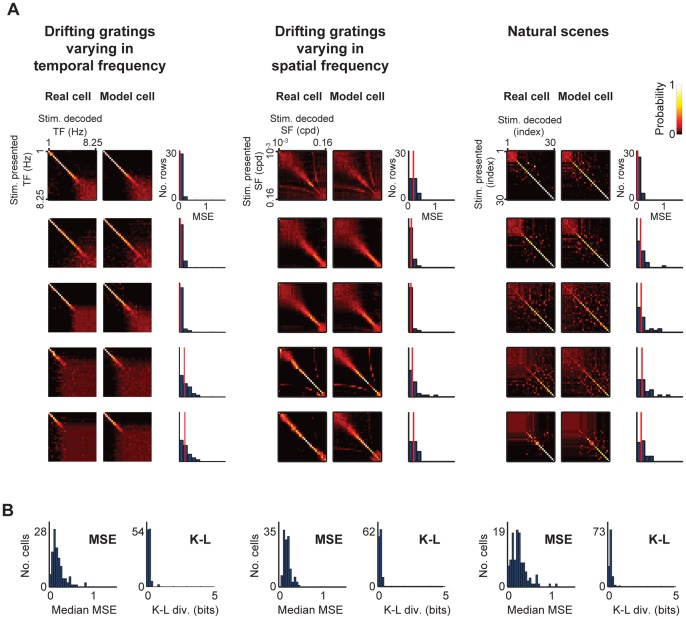
The posterior stimulus distributions of the model cells closely matched those of their real cell counterparts. (**A**) Pairs of matrices for each cell. The matrix on the left gives the posterior stimulus distributions for the real cell’s responses; the matrix on the right gives the same for the model cell’s responses. The histogram next to the pair gives a measure of the distance between them. Briefly, for each row, we computed the mean squared error (MSE) between the model’s posterior and the real cell’s posterior and then normalized it by dividing it by the MSE between the real cell’s posterior and a randomly shuffled posterior. A value of 0 indicates that the two rows are identical. A value of 1 indicates that they are as different as two randomly shuffled rows. Because of data limitation, occasional cells showed values higher than 1. The vertical red line indicates the median value of the histogram, the MSE α value. (**B**) Histogram of the MSE α values for all cells in the data set, and histogram of the K-L α values for all cells in the data set (*n* = 109, 120 and 113 cells for the stimuli, respectively). As shown in these histograms, most of the distances are low. For the MSE, the median α value is 0.21. As mentioned above, 0 indicates a perfect match between model and real responses, and 1 indicates correspondence no better than chance. For the K-L divergence, the median α value is 0.18. As a reference, 0 indicates a perfect match between model and real responses, and 4.9 bits (the stimulus entropy) indicates a poor match – this would be the K-L divergence between perfect decoding by a real cell and random decoding by a model cell. The complete set of matrices for the data set are provided in Figs. S1, S2, S3.

We then evaluated the quality of the information by plotting the posterior stimulus distributions produced by the model cells and comparing them to those produced by their real cell counterparts. The evaluation was performed using the same stimulus sets as used for the information analysis and is shown in Fig. 4. For each cell and its corresponding model, we plotted a matrix (see examples in Fig. 4A). The vertical axis of each matrix indicates the stimulus that was presented, and the horizontal axis indicates the average posterior stimulus distribution for that stimulus. For instance, in the top left matrix, when the grating with the lowest temporal frequency is presented (top row), the posterior is sharply peaked (as shown by the presence of a single bright spot in the row), indicating that the responses convey, with a high degree of certainty, that the presented grating must have been the lowest temporal frequency (since the peak occurs in the first column). In contrast, when the grating with the highest temporal frequency is presented (bottom row), the posterior has no sharp peak, but rather is distributed over many frequencies, as indicated by the wide red region; in this case, the responses provide little information about what the stimulus is - they convey only that the grating was in the high frequency range, but not which high frequency in particular.

For each pair of matrices (real and model), we quantified the distance between them in two ways. First, we performed a row-by-row comparison based on mean squared error (MSE); the row-by-row distance values for each example are shown in the histogram to the right of the matrices, with the median row distance, called the MSE α value, indicated by the red vertical line (see *Methods*). Second, we performed a row-by-row comparison based on Kullback-Leibler (K-L) divergence, and, likewise, took the median K-L divergence across rows, called the K-L α value. In Fig. 4B, we show the results for all the cells in the data set. For each stimulus type, two histograms are shown: the histogram on the left shows the MSE α values, and the histogram on the right shows the K-L α values. As shown (Fig. 4B), the vast majority of the distances are low, indicating that the vast majority of the cells have posteriors close to those of their real cell counterparts. (The complete set of matrices for the data set is provided in Figs. S1, S2, S3; for each cell, the matrices for both the model and the real cell are shown. In addition, we show in Fig. S5 analysis of the divergence between the posteriors produced by the models and those produced by the real cells using the Jensen-Shannon divergence, and the analysis leads to the same conclusion: the divergence is small: 0.14 (close to 0 on a 0–1 scale); see Fig. S5.).

The significance of Fig. 4 is two-fold: first, it shows that there are many different kinds of posterior stimulus distributions among the real cells, and, second, that the model cells accurately reproduce them. For example, some cells provide information about low frequencies, others provide information about high frequencies, or show complex patterns. But in nearly all cases, with several hundred cells examined, the behavior of the real cell is captured by the model. This provides strong evidence that the model cells can serve as proxies for the real cells and that the virtual retina approach is sound - i.e., the models can be used to gather data about what kinds of information the different ganglion cells carry, and these data will be reliable.

### Using the Virtual Retina Approach to Make Predictions

In the preceding section, we showed that the responses of the model cells match those of the real cells in both the quantity and the quality of the information they carry. Next, we took the analysis to the next level and used the match between model and real cells to make predictions about population coding. We made predictions at two levels - the level of ganglion cell recordings (electrophysiology) and the level of behavioral performance (optomotor tracking).

**Figure 5:**
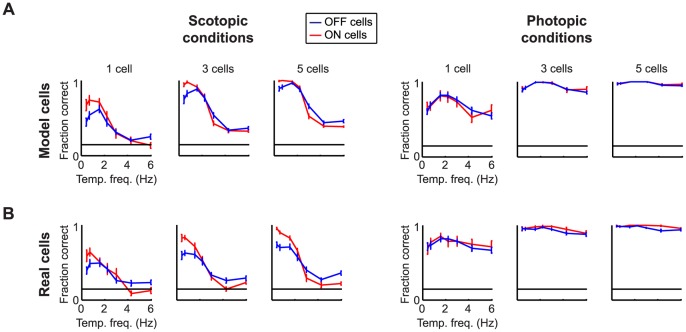
The model was able to make reliable predictions about the behavior of the real cell classes. Each plot shows the “fraction correct” as a function of temporal frequency for ON cells (*red*) and OFF cells (*blue*). *Top left*, the model indicates that ON cells are better at distinguishing among low temporal frequencies than OFF cells under scotopic conditions, whereas OFF cells are better at distinguishing among high temporal frequencies. *Bottom left*, the real cells indicate the same. *Top, looking across scotopic and photopic conditions*, the model indicates that these differences only occur under scotopic conditions. *Bottom, looking across scotopic and photopic conditions*, the real cells indicate the same. *Top, looking across the two conditions* ***,*** the model shows that ON and OFF cells perform well only for a narrow range of frequencies under scotopic conditions, but over a broad range under photopic conditions. *Bottom, looking across the two conditions,* this prediction held for the real cells as well. Predictions were made with increasing numbers of cells until there was indication of performance saturation. Error bars are SEM. The horizontal black line corresponds to performance at chance (7 stimuli, 1/7 correct).

We started with the electrophysiology predictions (Fig. 5). We set up a visual discrimination task: The model was first presented with stimuli - drifting gratings that varied in temporal frequency - and model responses were obtained. We then decoded the responses using maximum likelihood decoding (see *Methods*). On each trial of the task we asked: given the responses, what was the most likely stimulus, that is, the most likely frequency of the grating? Lastly, for all trials we tallied the fraction of times the responses provided the correct answer, the “fraction correct”. To make specific predictions about specific classes, we focused on two well-defined cell types, the ON and OFF transient cells, as these have been shown in the mouse retina to form statistically significantly distinct clusters [27]. We performed the task with populations made up exclusively of ON transient cells or exclusively of OFF transient cells. We also ran the task with models built both at scotopic and photopic light levels, since ganglion cells are known to behave differently under these conditions [28].

Several results emerged: The first was that the ON cells were better able to distinguish among low temporal frequencies than the OFF cells under scotopic conditions. The second was that the OFF cells were better able to distinguish among high temporal frequencies than the ON cells, also under scotopic conditions. The third was that these differences existed only under scotopic conditions: the two cell classes performed approximately equally well under photopic conditions. Finally, the last was that the ON and the OFF cells performed well only for a narrow range of frequencies under scotopic conditions, but over a broad range under photopic conditions.

We then tested the predictions: we presented the same stimuli to retinas on a multi-electrode array and recorded ganglion cell responses. We then decoded the real cell responses as we had decoded the model cell responses, i.e., using maximum likelihood. As shown in Fig. 5, the real cells behaved the same way as the model cells, thus indicating that the model cells served as reliable proxies for the real cells.

**Figure 6:**
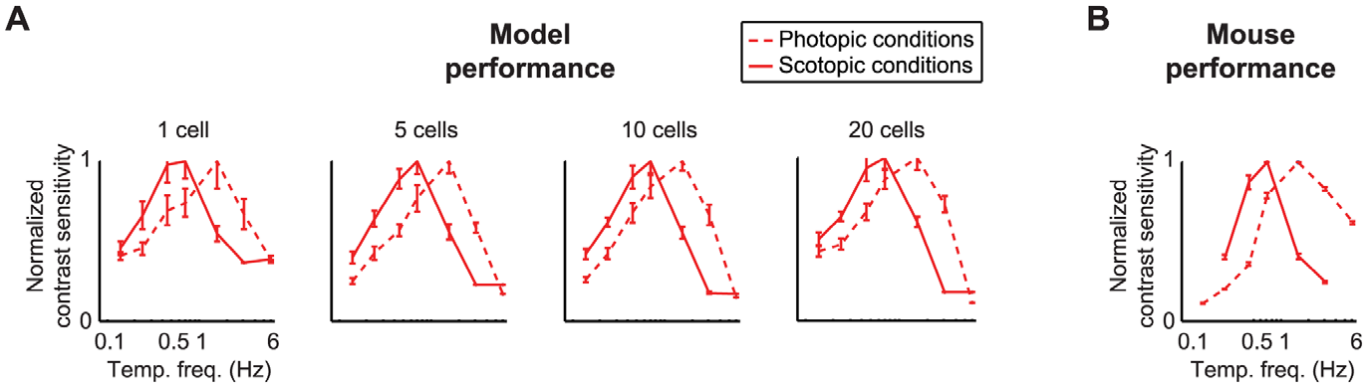
The model predicted the shift in optomotor performance. Each plot shows normalized contrast sensitivity under photopic and scotopic light conditions. (**A**) The model predicts a shift toward higher temporal frequencies as the animal moves from scotopic to photopic conditions, with the peak shifting from 0.7 Hz to 1.5 Hz. The prediction was robust from 1 cell to saturation (20 cells). (**B**) The animals’ behavioral performance shifted to higher temporal frequencies, as predicted (*n* = 5 animals). Error bars are SEM.

Finally, we advanced to predictions about behavior (Fig. 6). To do this, we used a standard optomotor task because it’s readily quantifiable and allows us to selectively probe the ON cell population, since only ON cells project to the accessory optic system (AOS), which drives the optomotor behavior [29], [30]. Thus, it eliminates the need to selectively inactivate OFF cells. With this task, the animal is presented with a moving grating of a particular temporal frequency. If the animal can see the grating, it tracks the grating’s motion; otherwise, it doesn’t. The animal is essentally asked: is the grating present or absent?

To make predictions about optomotor behavior, we asked the model the same question as we asked the animal: is the grating present or absent? To answer this, we used the same maximum likelihood decoding approach as we used in the electrophysiological experiments described above, except that instead of giving the decoder seven options to choose from, it was given two options, grating present or grating absent, to match the behavioral task. Finally, for both the behavior and the model performance, we measured contrast sensitivity, which is defined as the contrast at which 75% of the stimuli were correctly decoded, as is standard for two-alternative forced choice psychophysics [31].

As shown in Fig. 6A, the model cells predict that the tuning curves will shift to higher temporal frequencies under photopic conditions, with the predictions robust from 1 cell to 20 cells, where performance starts to saturate. Fig. 6B, which gives the behavioral performance, shows that this prediction is borne out. When optomotor performance was measured under scotopic conditions and under photopic conditions, with >3 hours in between to allow for adaptation to the light level, the same shift in tuning was observed – that is, the tuning curves obtained under photopic conditions were shifted toward higher temporal frequencies relative to those obtained from the same animals when they were performing the task under scotopic conditions (*n* = 5 animals). Thus, both the model and the behavior show that there is a shift in tuning that occurs as the animal moves from photopic to scotopic conditions – that is, the ganglion cells show a difference in their tuning properties, presumably to accommodate the different visual environments.

## Discussion

The visual system can represent a broad range of stimuli in a wide array of different conditions. A major contributing factor to this is the presence of different cell types with different visual sensitivities and response properties. Understanding the roles of the different cell types and how they work together has been a long-standing goal, one that has been difficult to advance because of the large space of possible stimulus/response relationships. Large data sets are needed to allow one to pull out patterns and extract rules about what the different cell types are doing.

Here we described a tool for addressing this. It’s a data-driven LN model of retinal input/output relationships. LN models have been used to address other population coding problems, in particular, the role of noise correlations [22]. Our aim here was to show that they can also serve another valuable function: they can serve as a tool for studying the contributions of the different retinal cell classes to the overall visual representation. Additionally, the LN models described here differ from other LN models in that they’re effective on a broad range of stimuli, allowing them to be used to study the roles of the different cell classes using natural scene stimuli in addition to artificial stimuli.

To demonstrate the effectiveness of the tool, we evaluated it three ways: first, we measured the amount of information the model cells carried compared to the amount carried by their real cell counterparts. The results showed that the model cells carried more than 90% of the information. Second, we assessed the quality of the information. For this, we evaluated the posterior stimulus distributions produced by the model cells and compared them to those produced by their real cell counterparts. The results showed, using both MSE and K-L divergence as measures, that the two closely matched: e.g., for >90% of the cells, the K-L divergences were less than 0.5 bits out of a 4.9 bit stimulus.

Lastly, we made predictions about the contributions of two ganglion cell types, ON transient and OFF transient cells, to the representation of motion stimuli (drifting gratings varying in temporal frequency) under different light conditions, and tested the predictions using both electrophysiological and behavioral experiments. The results show that the predictions were borne out; they add to the growing notion that the ON and OFF pathways are not simply mirror images, but in fact have distinct signaling properties, particularly with respect to the representation of motion information.

**Figure 7:**
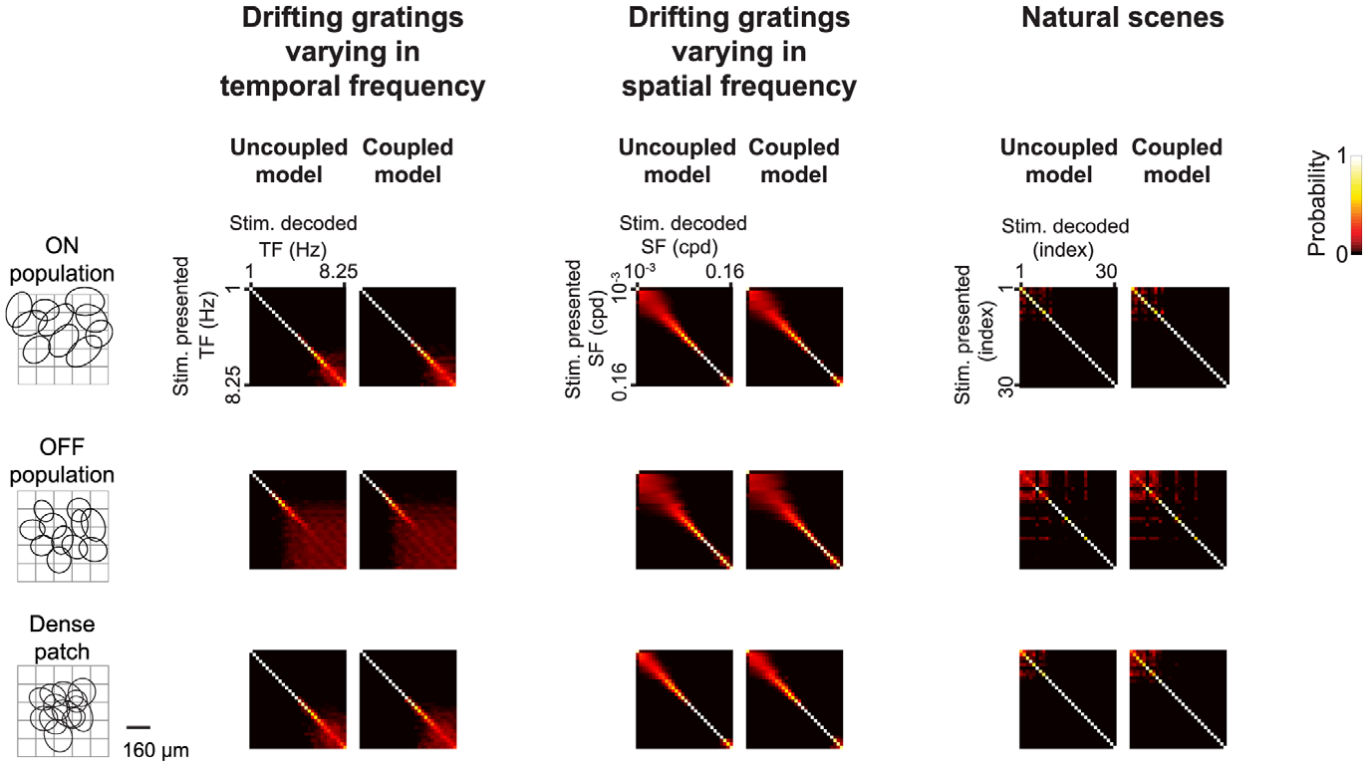
Posterior stimulus distributions generated using models that included correlations among the ganglion cells and models that treated the ganglion cells as independent. The posterior stimulus distributions (matrices) were calculated using three populations of cells: a patch of transient ON cells (*n = *10 cells), a patch of transient OFF cells (*n = *11 cells), and a patch that included all the cells recorded in a local region of the retina (*n = *12 cells). For each population, models were built without correlations (*left*) and with correlations (*right*) included. Distances between coupled and uncoupled posteriors were very low: MSE α values were below 0.05, and K-L α values were below 0.1 bits. To avoid data limitation, response distributions for the population were taken directly from the analytical form of the model, as in [22], [23].

Note that the results in Figs. 1, 2, 3, 4, 5, 6 treat ganglion cells as conditionally independent. In principle, though, the virtual retina approach can include noise correlations among cells, for example, by including coupling currents [22]. From the recordings we presented here, in addition to fitting the independent cell models, we also fit coupled models for several patches of ganglion cells and compared the coupled populations’ performance on natural scenes and drifting gratings to that of the independent models. We found that, for the three stimulus sets tested, including coupling in the models had little effect on the quality of the information conveyed by the population (Fig. 7). This suggests that using a library of conditionally independent virtual cells is an effective and practical approach for the purposes of making predictions about population coding.

### Decoding Versus Encoding

**Figure 8:**
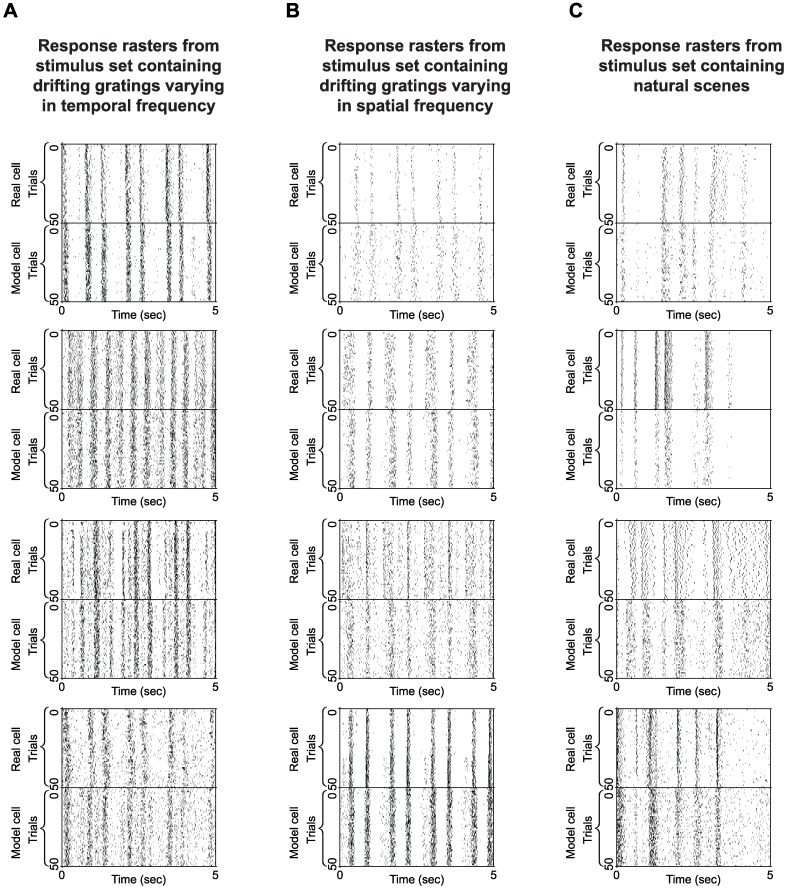
Response rasters for the first 12 cells shown in Fig. 4. Fig. 4 shows real and model cell performance using three sets of stimuli. Here we show rasters of the underlying responses. **A**. Rasters for the responses to the drifting gratings that varied in temporal frequency. The stimulus is a continuous stream of drifting gratings with uniform gray fields interleaved (grating stimuli are 1 s; gray fields are 0.33 s). 5 s of a 41 s stimulus are shown (repeated 50 times). Note that each cell is viewing a different location in the movie. **B**. Rasters for the responses to the drifting gratings that varied in spatial frequency. As above, the stimulus is a continuous stream of drifting gratings with uniform gray fields interleaved (grating stimuli are 1 s, gray fields are 0.33 s). Each cell is viewing a different location of the stimulus. **C.** Rasters for the responses to the natural scene movies. The stimulus is a continuous stream of natural movies with uniform gray fields interleaved (natural movie snippets are 1 s long, gray fields are 0.33 s). Note again that each cell is viewing a different location in the movie: this is most notable in the rasters for the natural scene snippets, since these are not periodic stimuli.

This paper focuses on decoding – on using this LN model as a tool to understand how populations of neurons collectively represent visual scenes, including spatio-temporally varying natural scenes. The model also works well for encoding as shown in ref. 26, where we used the model as the underpinning for a retinal prosthetic, a context in which encoding is the key issue. In ref. 26 rasters demonstrating encoding performance using a broad range of natural stimuli are shown (both in the main text and Supp. Info.). Here, for the convenience of the reader, we show rasters for all the cells used in this study: in Fig. 8, we show raster plots for the first 12 cells whose average posteriors are shown in Fig. 4, and, in Supp Info, we show the rasters for every cell in the data set (>300 cells) (Figs. S7, S8, and S9), just as we show the average posteriors for every cell (Figs. S1, S2, and S3). Note that for clarity, the raw data is given (the rasters and average posteriors) rather than, for example, the *r* ^2^ values, since *r* ^2^ has well-known pitfalls for measuring encoding (e.g., sensitivity to small time shifts).

In sum, ref 26 shows how the model works for practical applications, such as building a retinal prosthetic, and in this paper, we show that it serves as a powerful tool for basic science, too: to address questions in population coding.

### Conclusion

A major stumbling block in studying high-dimensional problems, such as population coding with complex, heterogeneous populations, is that a considerable amount of exploratory research is needed. Exploratory research in live experiments is very slow and requires large numbers of animals. If one has a reliable model for the population, this problem can be greatly ameliorated. The model described here, the virtual retina, provides a way to rapidly probe the roles of the different cell classes in the ganglion cell population, guiding dimension reduction and the development of population coding hypotheses, which can then be tested and verified in the lab.

## Methods

### Recording

Electrophysiological recordings were obtained from the isolated mouse retina. Recordings of central retinal ganglion cells (RGCs) were performed on a multielectrode array using methods described previously [21], [32]. Spike waveforms were recorded using a Plexon Instruments Multichannel Neuronal Acquisition Processor (Dallas, TX). Standard spike sorting methods were used as in ref. [21]. Cells that did not have a clear spike-triggered average (STA) or that had excessive refractory period violations (>2%) were discarded. All procedures on experimental animals were carried out under the regulation of the Institutional Animal Care and Use Committee (IACUC) of the Weill Cornell Medical College of Cornell University and in accordance with NIH guidelines; IACUC protocol number 0807–769A, approved July 25, 2011.

### Constructing the Models

Each ganglion cell’s stimulus/response relationship was modeled using a linear-nonlinear-Poisson structure (for general review, see refs. [23] and [24]). For producing a linear-nonlinear cascade that’s effective on a broad range of stimuli, we used the approach described in refs. [25] and [26], which focus on developing models for retinal prosthetics. Briefly, for each cell, the firing rate, 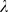, elicited by the stimulus *s* at time *t* is given by
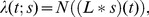
(1)


where *L* is a linear filter corresponding to the cell’s spatio-temporal impulse response, *N* is a function that describes its nonlinearity, and * denotes spatio-temporal convolution. The firing rate, 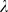, was then used as the intensity of an inhomogeneous Poisson process. Each cell’s linear filter was assumed to be a product of a spatial function (on a 10×10 array of pixels, centered on the cell’s receptive field); and a temporal function (18 time bins, 67 ms each, total duration 1.2 s). Dimensionality was reduced by assuming the temporal function to be a sum of 10 basis functions (raised cosines), as in Nirenberg and Pandarinath [26], following Pillow et al. [22]. The nonlinearities *N* were parameterized as cubic spline functions with 7 knots. Knots were spaced to cover the range of values given by the linear filter output of the models. Parameters were determined by maximizing the log likelihood of the observed spike trains under the model, averaged over all stimuli *s* (stimuli are given below under *Stimuli)*:
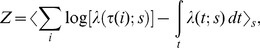
(2)


where 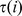 is the time of the *i*th spike, and *t* ranges over all time for the stimuli used for the fitting. To carry out the maximization, we began by assuming that the nonlinearity *N* was exponential, since in this case, the log likelihood *Z* (eq. 2) has one global maximum [23]. After optimizing the linear filters for an exponential nonlinearity (by coordinate ascent), the nonlinearity was replaced by a spline. Final model parameters were then determined by alternating stages of maximizing the log likelihood with respect to (i) the spline coefficients and (ii) the filter parameters, until a maximum was reached. For the data in Figs. 1, 2, 3, 4, 5, 6, the cells were modeled as conditionally independent; for the data in Fig. 7, correlations among the cells were taken into account, as in ref. [22].

### Stimuli

Two stimuli were used to generate the models: binary spatio-temporal white noise and a natural scene movie; the reasoning behind this is described in detail in ref. [25], p. 30–32, and also ref [26]. Both were presented at a frame rate of 15 Hz, and had the same mean luminance and contrast (mean luminance was 1.7 µW/cm^2^ at the retina (measured using a New Focus Model #3803 Power Meter (San Jose, CA)); RMS contrast was 0.27 µW/cm^2^). The natural scene movie had a temporal power spectrum of 1/*f* ^2.04^, where *f* is temporal frequency, and a spatial power spectrum of 1/*w* ^2.09^, where *w* is spatial frequency.

Three sets of stimuli were used to test the model: drifting sine-wave gratings that varied in temporal frequency (the range was 1 to 8.25 Hz, to cover the range to which mouse retinal ganglion cells are sensitive [33]; the spatial frequency was fixed at 0.058 cycles per degree); drifting gratings that varied in spatial frequency (the range was 0.0012 to 0.155 cycles per degree, again, chosen to cover the range to which mouse retinal ganglion cells are sensitive [34], [35]; temporal frequency was 2 Hz); and a set of natural scene movies (different from the movie used to build the models, but taken under the same conditions, i.e., same mean luminance and contrast). All stimuli used for testing the model (gratings and natural movies) were presented in 1 s segments, interleaved with 333 ms segments of uniform gray. Each of these segments was considered to be a separate stimulus for the mutual information calculations, described below.

### Calculation of Mutual Information

Mutual information *I* between a set of stimuli, *s*, and a set of responses, *r*, was calculated using the standard expression,
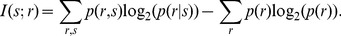
(3)


To estimate the probabilities in eq. 3, we first binned responses into time bins of length 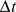, so that each response could be represented as a vector of spike counts, 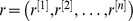. We then treated the number of spikes in a bin as a Poisson random variable 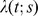, with mean rate estimated from the binned responses:
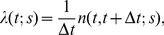
(4)


where 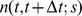 denotes the average spike count in a bin from time *t* to time 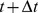, in response to the stimulus *s*. The probabilities required by eq. 3 can now be written in terms of the Poisson intensities 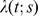 by
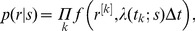
(5)


where 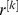 is the number of spikes in the *k*th bin, 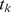 is the time of the *k*th bin, and 
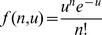
 is the probability density function for the Poisson distribution of mean *u* [36]. Calculations were performed in parallel for several bin widths (4, 8, 16, or 32 bins per 1 s stimulus segment).

To complete the calculation of mutual information, the equation for the conditional response probability (eq. 5) is substituted into eq. 3. The summation over all possible responses indicated in eq. 3 corresponds to sums over all possible values of the spike counts in each bin, 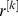. This sum was estimated by a Monte Carlo procedure, by drawing stimulus and response pairs based on their probabilities.

To check the robustness of the information calculations, we also used an alternate procedure that doesn’t rely on treating spike counts as Poisson random variables (Fig. S4). In this procedure, we take the observed spike counts (model or real) in each time bin and divide them into a number of response levels (quartiles). We then count the number of times a given level occurred in a given time bin, i.e., we obtain the probability that a response level in a bin falls into one of the quartiles. As shown in *Supporting Information,* Fig. S4C, the results are very close to those determined with the Poisson approach. (Note that the reason we chose four response levels is because four levels captured the complete response distribution, or very close to the complete response distribution: i.e., for 63 ms bins and 31 ms bins, >95% of bins had at most 3 spikes, so using four response levels (0, 1, 2, or 3 or more spikes) was essentially the same as using spike counts.) Note also that for Fig. S4C, information was calculated using debiasing, following the quadratic extrapolation method in ref. [37]; this was done because the quartile method requires the estimation of multiple response probabilities, raising the possibility of data limitation. For completeness, we also performed a comparable debiasing for the Poisson approach (Fig. S4B). As shown in the figure, the information results are robust to the differences in the different methods of analysis.

### Posterior Stimulus Distributions

Posterior stimulus distributions, 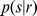, were used to quantify and visualize the type of information (the quality of the information) transmitted by the model cells and by their corresponding real cells. These distributions were summarized in the form of a matrix 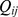, which gives the average posterior probability 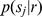 of each stimulus *s_j_*, calculated over all *N_trials_* trials (*N_trials_*  = 25) in which the stimulus presented is *s_i_*:
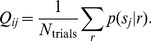
(6)


To calculate 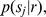 we used Bayes’ Theorem:
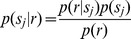
, where 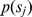 was uniform across all stimuli. We used uniform priors, since it puts the greatest emphasis on the cell’s responses; thus, it’s the most conservative test. The term 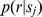, the probability that response, *r*, was elicited by another stimulus, *s_j_*, was calculated as in eq. 5.

To determine the accuracy of the model’s average posterior matrix 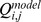, we compared it row-by-row with the actual average posterior matrix 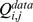 via a mean squared error (MSE),
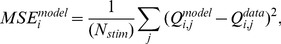
(7)


where 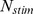 is the number of stimuli (*N_stim_* = 30). To gauge the departure of the MSE from 0, we compared it to the average MSE obtained from matrices generated by shuffling the elements of 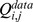 randomly within each row. This yields an index
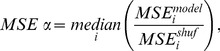
(8)


where 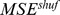 is an average over quantities similar to eq. 7, with shuffled rows taking the place of the model average posterior row. Thus, 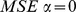 indicates that the model average posterior (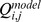) perfectly reproduced the one calculated from the actual responses (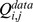), and 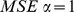 indicates that this correspondence was no better than the correspondence between a shuffled model row and the actual responses.

In addition to MSE, we quantified the accuracy of the model posteriors using Kullback-Leibler (K-L) divergence, a standard comparison for probability distributions. For each row *i* of a model cell’s posterior matrix 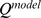 and its corresponding real cell’s posterior matrix 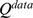, we first regularized both rows using the standard method, adding 0.5 to each posterior stimulus bin *j* [38]:
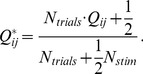
(9)


We then calculated the K-L divergence of the model row with the real row, using the standard equation [39], and took the median of these row-by-row divergences to get the K-L α value:
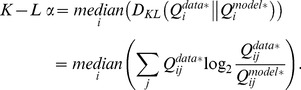
(10)


See Fig. S5 for analysis using a measure that does not require regularization, the Jensen-Shannon divergence, and see Fig. S6 for analysis using the K-L divergence, but calculated using half the data, the latter showing that the conclusions of the analysis using this measure were robust to data limitation.

### Testing Predictions using Electrophysiological Experiments

As described in *Results*, we measure the models’ ability to make reliable predictions about the behavior of different cell classes by comparing the performance of real and model cells in the context of a forced-choice stimulus identification task [40]. Specifically, we performed a maximum likelihood decoding of responses to a stimulus, using either real or model responses, and determined the fraction of decoding trials that resulted in correct stimulus identifications, the “fraction correct”.

To calculate the fraction correct, we examined population responses *r* to each stimulus *s_i_* and tallied each population response as a correct trial if the stimulus *s_i_* was the most likely stimulus to elicit it. As before, the posterior probabilities 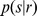 were calculated from the conditional probabilities 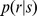 via Bayes’ Theorem. When *r* includes responses from multiple cells (i.e., when multiple cells were used to decode), the cells’ response probabilities were treated as conditionally independent [32], [41], [42], so that 
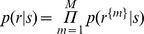
, where *r^{m}^* is the response of cell *m*, and *M* is the total number of cells in the population. As with calculating posteriors, we split the data in half, and used the training half to estimate 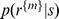 using eq. 5.

For this analysis, models were fit, and analysis was performed under two light conditions (photopic conditions, with a mean light intensity of 0.25 µW/cm^2^, and scotopic conditions, with a mean light intensity of 2.8×10^−5 ^µW/cm^2^). The same set of retinal ganglion cells were used in both conditions to allow comparison across the conditions. The stimulus was a set of drifting sine-wave gratings at 7 different temporal frequencies, ranging from 0.44 Hz to 6 Hz, with a spatial frequency of 0.039 cycles/deg.

### Testing Predictions Using Behavioral Experiments

As described in *Results*, we measure the models’ ability to make reliable predictions about animal behavior by comparing model predictions with behavioral performance on a standard optomotor task. Behavioral responses were measured as in ref. [40]. Briefly, the animal was placed in a virtual reality chamber. A video camera, positioned above the animal, provided live video feedback of the testing chamber. The animal was assessed for tracking behavior in response to stimuli that were projected onto the walls of the chamber. The stimuli were drifting sine-wave gratings that varied in temporal frequency and contrast (temporal frequency ranged from 0.14 to 6 Hz, contrast ranged from 100% to 1%); spatial frequency, as measured from the animal’s viewing position, was held fixed at 0.128 cycles/degree. On each trial of the task, the animal either tracked it or failed to track the stimulus, measured as in ref. [40]. The contrast threshold for each stimulus was then determined by identifying the lowest contrast for which 75% of trials were correct, as is standard for two-alternative forced choice psychophysics [31].

For the model, we used the same maximum likelihood approach as in the electrophysiological experiments described above, except, instead of seven options (seven different frequencies), we decoded each grating response using two options -grating present or grating absent - to match the behavioral choice. Contrast threshold for each grating was then determined by identifying the lowest contrast for which 75% of trials were correct, as for the real behavior experiment.

As with the electrophysiology experiments, two sets of models were constructed, one under photopic conditions and one under scotopic conditions (the same light intensities were used).

## Supporting Information

Figure S1 **The complete set of posterior stimulus distributions (matrices) when the stimulus set consisted of drifting gratings that varied in temporal frequency; this is the complete set referred to in** **Fig. 4** **, left column (** ***n*** **= 109 cells).**
(PDF)

Figure S2 **The complete set of posterior stimulus distributions (matrices) when the stimulus set consisted of drifting gratings that varied in spatial frequency; this is the complete set referred to in** **Fig. 4** **, middle column (** ***n*** **= 120 cells).**
(PDF)

Figure S3 **The complete set of posterior stimulus distributions (matrices) when the stimulus set consisted of natural scene movies; this is the complete set referred to in** **Fig. 4** **, right column (** ***n*** **= 113 cells).**
(PDF)

Figure S4 **Robustness of the mutual information calculation.** Here we compare the mutual information between stimulus and response as calculated in the main text, with two alternative approaches. As in Fig. 1, 2, 3, we calculated the mutual information between each model’s responses and the stimulus and plotted it against the mutual information between the real cell’s responses and the stimulus; the calculations here were performed with the same cells and stimuli. Information was calculated at four bin sizes, from 250 to 31 ms. **(A)** Mutual information calculated by treating the spike count in each time bin as a Poisson random variable, as in Fig. 1, 2, 3. **(B)** The same calculation, but now debiased with quadratic extrapolation, as in [37]. **(C)** Mutual information calculated by taking observed (real or model) spike counts and dividing the counts into quartiles (see Methods). The spike count in each time bin was treated as a multinomial distribution, determined by calculating the empirical probability of observing each response level in the time bin. The quadratic extrapolation debiasing procedure of [37] was applied.
(PDF)

Figure S5 **The posterior stimulus distributions of the model cells closely matched those of their real cell counterparts, as measured using the Jensen-Shannon (J-S) divergence.** The figure shows histograms of the J-S α values for all cells in the data set (n = 109, 120 and 113 cells for the three sets of stimuli, respectively). Briefly, as described in Methods, the α value is the median of the row-by-row divergences for a pair of matrices, where one matrix is produced by the model, and the other is produced by the real cell. The J-S divergences are calculated from the “plug-in” estimator, without regularization (because regularization is not required). The median α value across all stimulus sets is 0.14 (close to 0 on a 0 to 1 scale). Greater than 90% of the α values are less than 0.22. (Note that the J-S divergence is on a different scale from the K-L divergence, even though they’re both in bits. For the J-S divergence, the scale is from 0 to 1 (the maximum J-S divergence is 1). For the K-L divergence, we used 0 to 4.9, since 4.9 bits is the stimulus entropy. For more detailed discussion of scale, see Fig. 4 legend.)
(PDF)

Figure S6 **Estimates of the Kullback-Leibler (K-L) divergence are not data-limited.** This figure is a scattergram of the α values across all experiments, calculated as in the main text. The α values calculated from half the trials are plotted against the α values calculated from all the trials. As shown, the points lie close to the line of identity.
(PDF)

Figure S7 **Raster plots for all cells viewing the stimulus set consisting of drifting gratings that varied in temporal frequency (** ***n*** **= 109 cells).** The stimulus is a continuous stream of drifting gratings with uniform gray fields interleaved (grating stimuli are 1 s, gray fields are 0.33 s). 5 s of a 41 s stimulus is shown (repeated 50 times). The vertical axis indicates the trials; 0 to 50 trials are shown for the real cell, followed by 0 to 50 trials for the model cell, following the layout in Figure 8 in the main text. The order of the rasters corresponds to the order of the posteriors in Figure S1.
(PDF)

Figure S8 **Raster plots for all cells viewing the stimulus set consisting of drifting gratings that varied in spatial frequency (** ***n*** **= 120 cells).** The stimulus is a continuous stream of drifting gratings with uniform gray fields interleaved (grating stimuli are 1 s, gray fields are 0.33 s). 5 s of a 41 s stimulus is shown (repeated 50 times). The vertical axis indicates the trials; 0 to 50 trials are shown for the real cell, followed by 0 to 50 trials for the model cell, following the layout in Figure 8 in the main text. The order of the rasters corresponds to the order of the posteriors in Figure S2.
(PDF)

Figure S9 **Raster plots for all cells viewing the stimulus set consisting of natural scenes (** ***n*** **= 113 cells).** The stimulus is a continuous stream of natural movies with uniform gray fields interleaved (natural movies are 1 s long, gray fields are 0.33 s). Note that each cell is viewing a different location in the movie: this is the case for the cells in Figs. S7 and S8 as well, but it is most obvious here in the natural scene rasters, since the movie is not a periodic stimulus. 5 s of a 41 s stimulus is shown (repeated 50 times). The vertical axis indicates the trials; 0 to 50 trials are shown for the real cell, followed by 0 to 50 trials for the model cell, following the layout in Figure 8 in the main text. The order of the rasters corresponds to the order of the posteriors in Figure S3.
(PDF)
