# Supplementary material for: A Virtual Retina for Studying Population Coding
Source: PLoS One. 2013 Jan 14;8(1):e53363. doi: 10.1371/journal.pone.0053363 (PMC3544815; doi:10.1371/journal.pone.0053363)
Supplement: Figure S1 — The complete set of posterior stimulus distributions (matrices) when the stimulus set consisted of drifting gratings that varied in temporal frequency; this is the complete set referred to in Fig. 4 , left column ( n = 109 cells). (PDF) [file pone.0053363.s001.pdf]

Figure S1

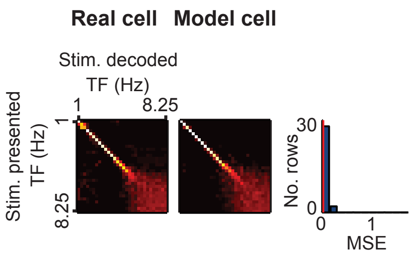

Complete set of posterior matrices using drifting gratings varying in temporal frequency (n=109 cells). Axes are labeled as in Fig. 4 (see example, top left).

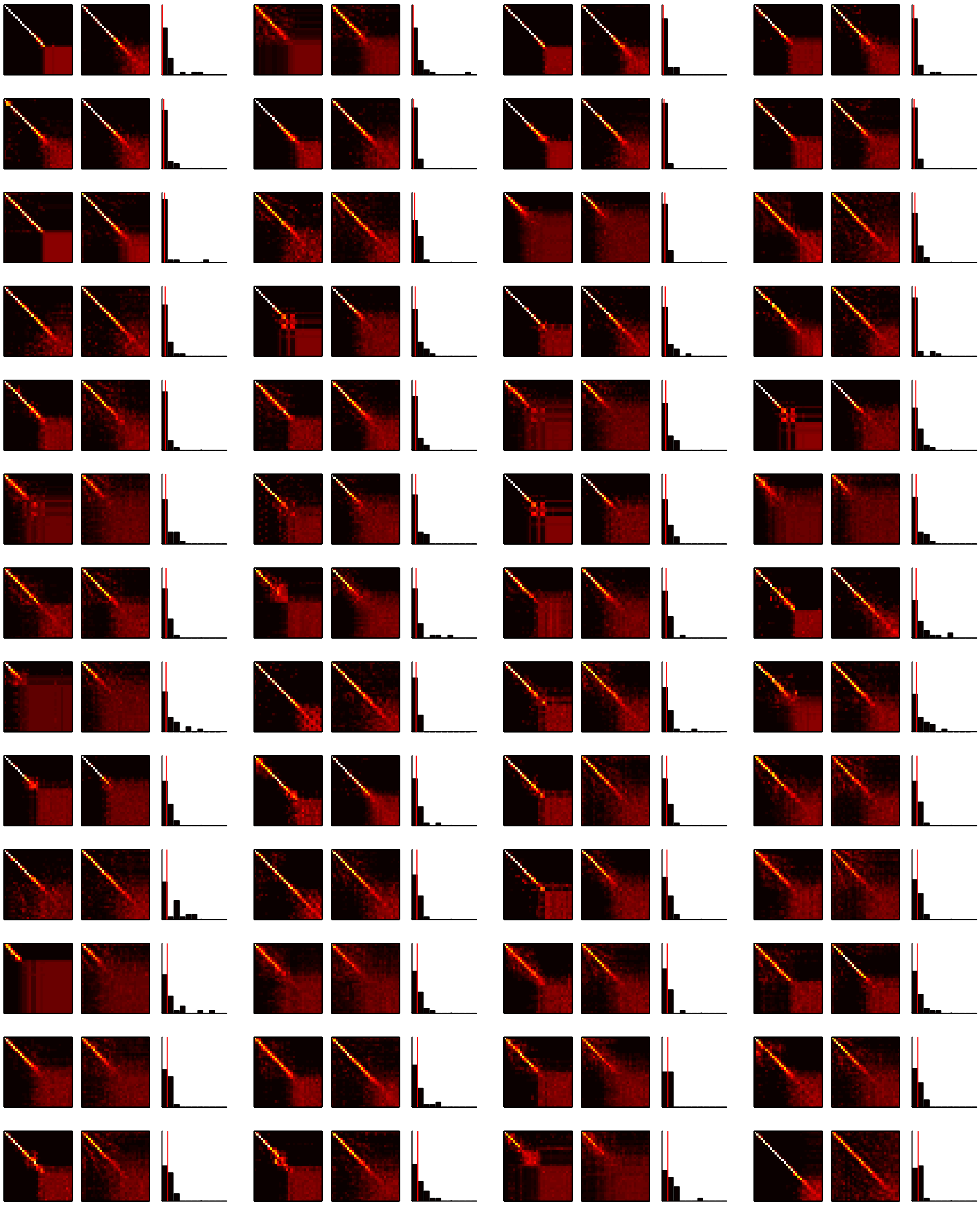

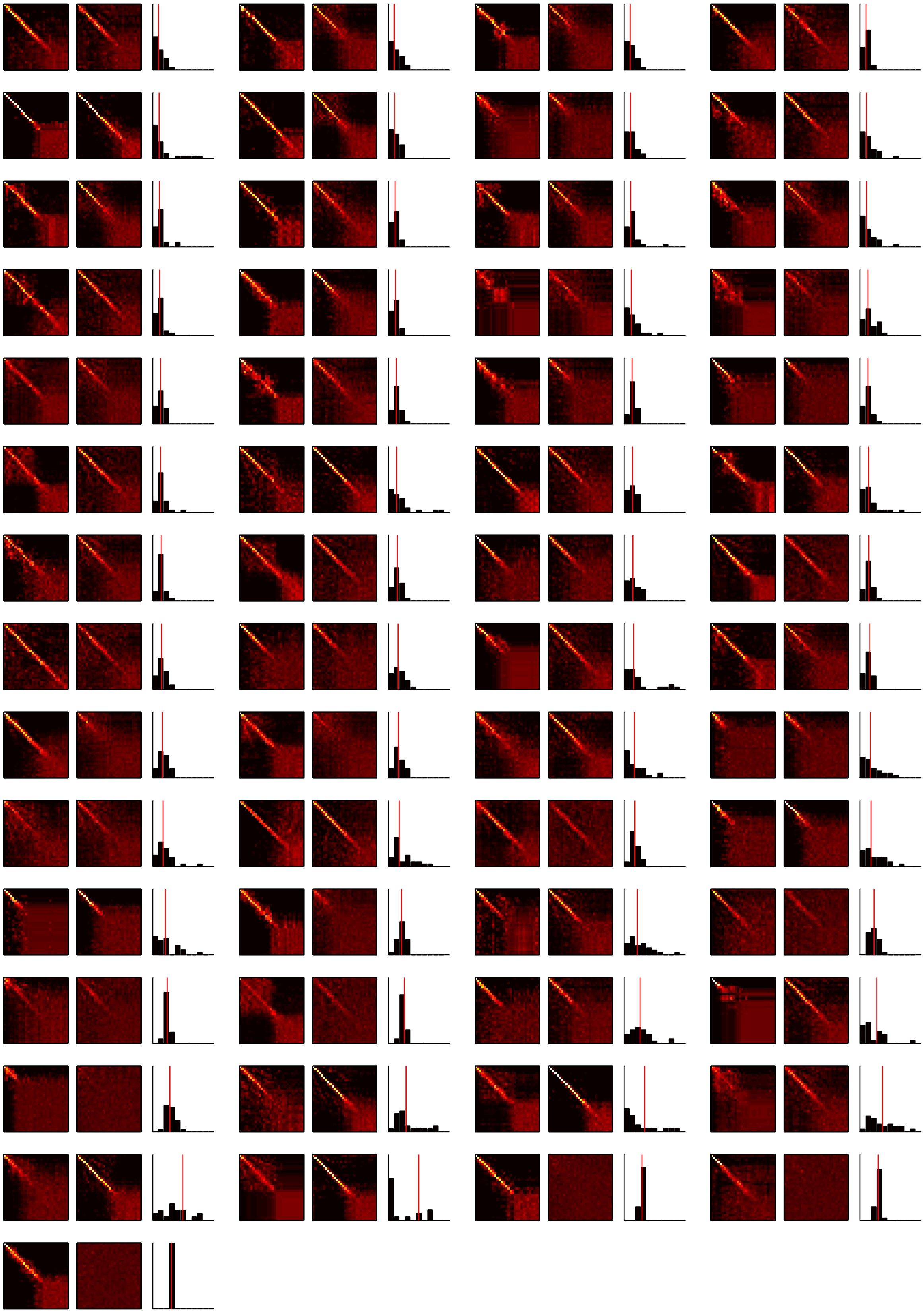

Figure S1. The complete set of posterior stimulus distributions (matrices) when the stimulus set consisted of drifting gratings that varied in temporal frequency; this is the complete set referred to in Fig. 4, left column (*n*=109 cells).
