# Supplementary material for: A Virtual Retina for Studying Population Coding
Source: PLoS One. 2013 Jan 14;8(1):e53363. doi: 10.1371/journal.pone.0053363 (PMC3544815; doi:10.1371/journal.pone.0053363)
Supplement: Figure S3 — The complete set of posterior stimulus distributions (matrices) when the stimulus set consisted of natural scene movies; this is the complete set referred to in Fig. 4 , right column ( n = 113 cells). (PDF) [file pone.0053363.s003.pdf]

Figure S3

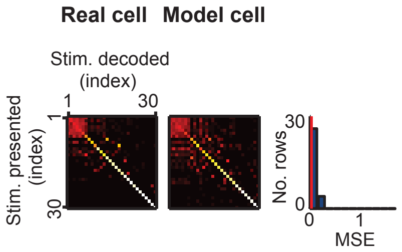

Complete set of posterior matrices using natural scene stimuli (n=113 cells). Axes are labeled as in Fig. 4 (see example, top left).

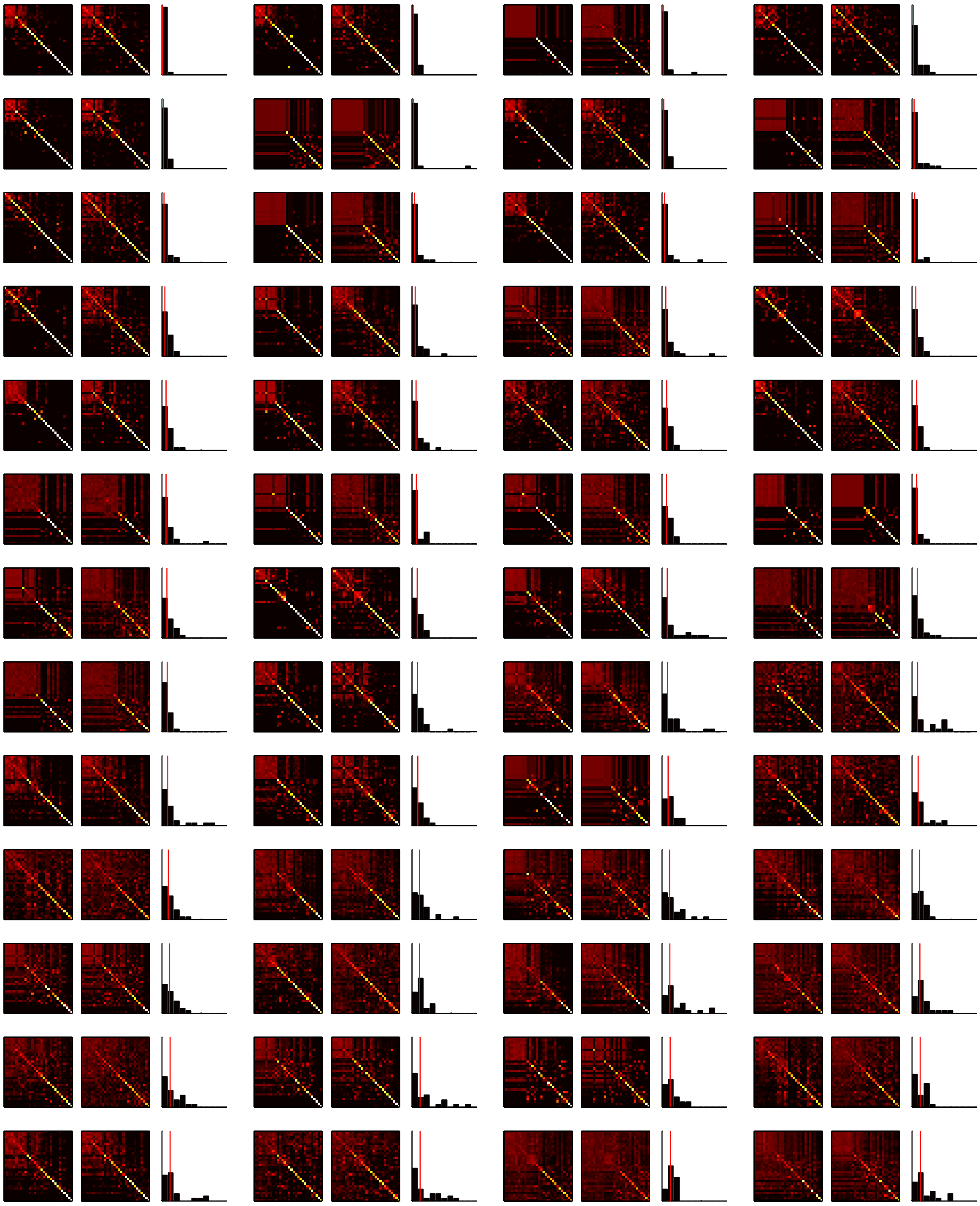

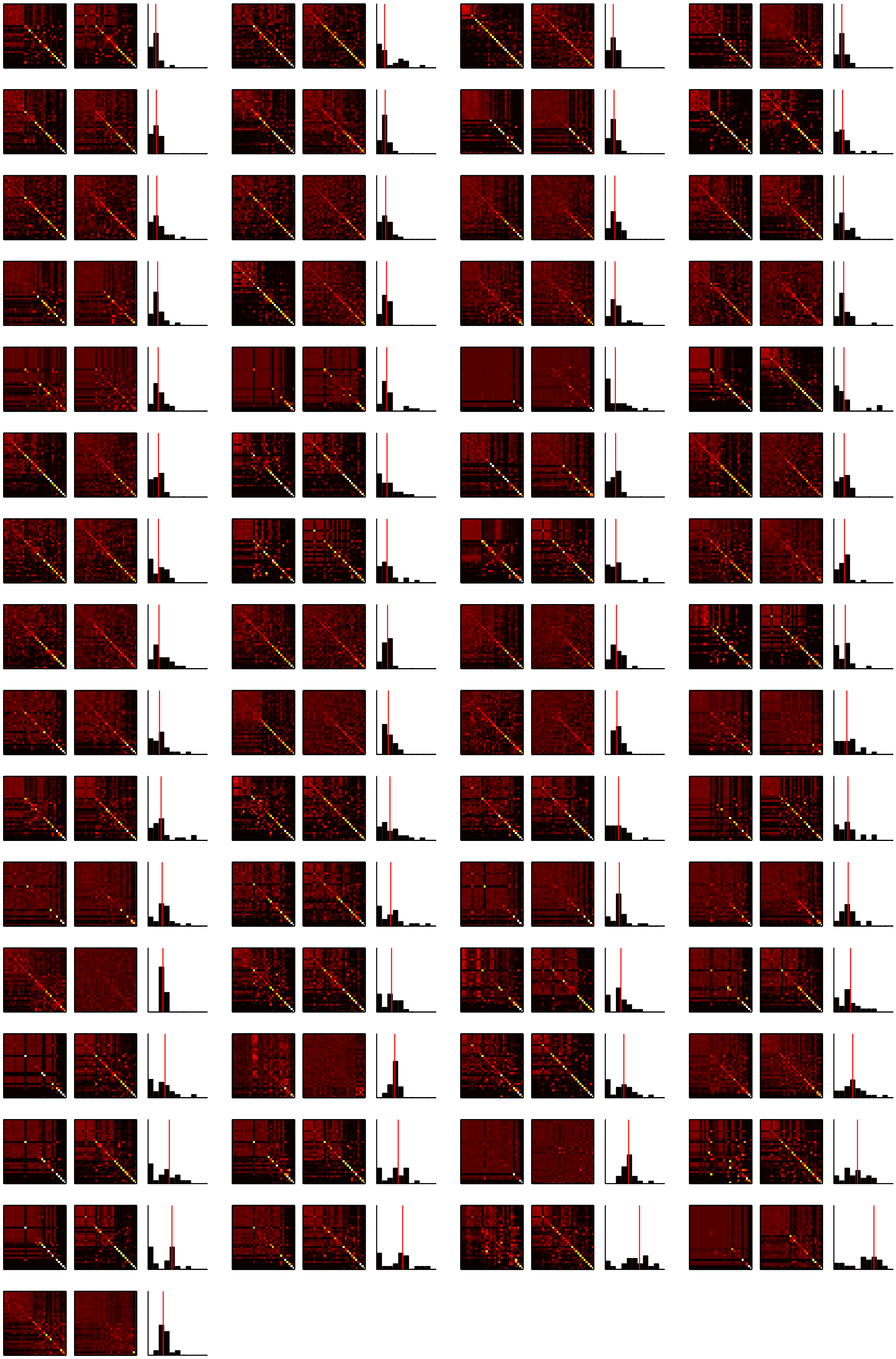

Figure S3. The complete set of posterior stimulus distributions (matrices) when the stimulus set consisted of natural scene movies; this is the complete set referred to in Fig. 4, right column (*n*=113 cells).
