# Supplementary material for: A Virtual Retina for Studying Population Coding
Source: PLoS One. 2013 Jan 14;8(1):e53363. doi: 10.1371/journal.pone.0053363 (PMC3544815; doi:10.1371/journal.pone.0053363)
Supplement: Figure S4 — Robustness of the mutual information calculation. Here we compare the mutual information between stimulus and response as calculated in the main text, with two alternative approaches. As in Fig. 1, 2, 3, we calculated the mutual information between each model’s responses and the stimulus and plotted it against the mutual information between the real cell’s responses and the stimulus; the calculations here were performed with the same cells and stimuli. Information was calculated at four bin sizes, from 250 to 31 ms. (A) Mutual information calculated by treating the spike count in each time bin as a Poisson random variable, as in Fig. 1, 2, 3. (B) The same calculation, but now debiased with quadratic extrapolation, as in [37]. (C) Mutual information calculated by taking observed (real or model) spike counts and dividing the counts into quartiles (see Methods). The spike count in each time bin was treated as a multinomial distribution, determined by calculating the empirical probability of observing each response level in the time bin. The quadratic extrapolation debiasing procedure of [37] was applied. (PDF) [file pone.0053363.s004.pdf]

Figure S4

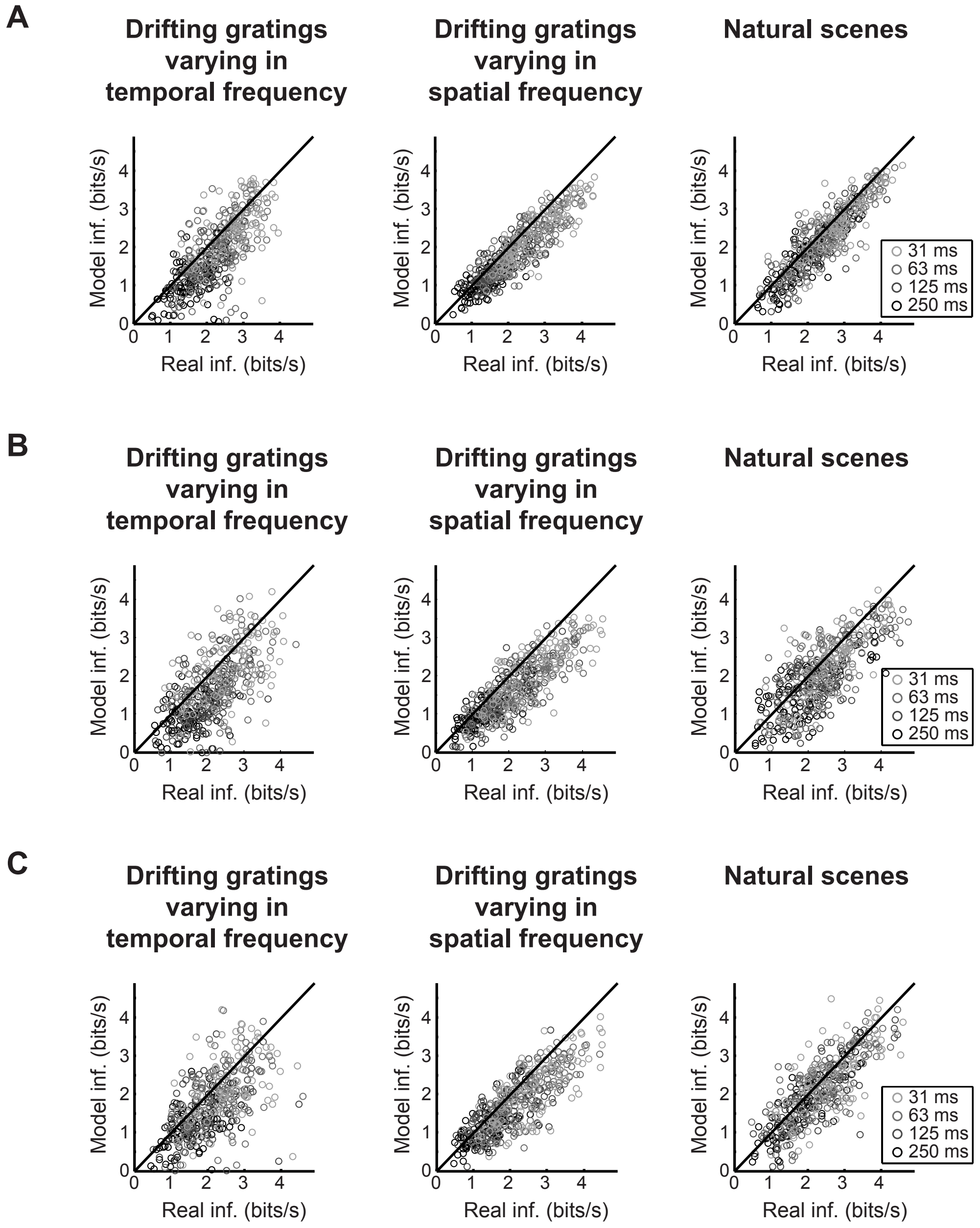

Figure S4. Robustness of the mutual information calculation. Here we compare the mutual information between stimulus and response as calculated in the main text, with two alternative approaches. As in Fig. 1–3, we calculated the mutual information between each model's responses and the stimulus and plotted it against the mutual

information between the real cell's responses and the stimulus; the calculations here were performed with the same cells and stimuli. Information was calculated at four bin sizes, from 250 to 31 ms. **(A)** Mutual information calculated by treating the spike count in each time bin as a Poisson random variable, as in Fig. 1–3. **(B)** The same calculation, but now debiased with quadratic extrapolation, as in [37]. **(C)** Mutual information calculated by taking observed (real or model) spike counts and dividing the counts into quartiles (see Methods). The spike count in each time bin was treated as a multinomial distribution, determined by calculating the empirical probability of observing each response level in the time bin. The quadratic extrapolation debiasing procedure of [37] was applied.
