# Supplementary material for: A Virtual Retina for Studying Population Coding
Source: PLoS One. 2013 Jan 14;8(1):e53363. doi: 10.1371/journal.pone.0053363 (PMC3544815; doi:10.1371/journal.pone.0053363)
Supplement: Figure S5 — The posterior stimulus distributions of the model cells closely matched those of their real cell counterparts, as measured using the Jensen-Shannon (J-S) divergence. The figure shows histograms of the J-S α values for all cells in the data set (n = 109, 120 and 113 cells for the three sets of stimuli, respectively). Briefly, as described in Methods, the α value is the median of the row-by-row divergences for a pair of matrices, where one matrix is produced by the model, and the other is produced by the real cell. The J-S divergences are calculated from the “plug-in” estimator, without regularization (because regularization is not required). The median α value across all stimulus sets is 0.14 (close to 0 on a 0 to 1 scale). Greater than 90% of the α values are less than 0.22. (Note that the J-S divergence is on a different scale from the K-L divergence, even though they’re both in bits. For the J-S divergence, the scale is from 0 to 1 (the maximum J-S divergence is 1). For the K-L divergence, we used 0 to 4.9, since 4.9 bits is the stimulus entropy. For more detailed discussion of scale, see Fig. 4 legend.) (PDF) [file pone.0053363.s005.pdf]

Figure S5

Measuring the distance between the average posterior stimulus distribution for the model cell and the real cell.

In the main text, we used mean squared error (MSE) and Kullback-Leibler (K-L) divergence to measure the distance between the average posterior stimulus distribution for the model cell and the real cell. Here we use an additional measure, the Jensen-Shannon (J-S) divergence. As shown (Fig. S5), it leads to the same conclusions as found in the main text: the average posterior distributions determined from the model cells closely match those determined from the real cells: as shown in the figure, the median α value across all stimulus sets is 0.14 (close to 0 on a 0 to 1 scale).

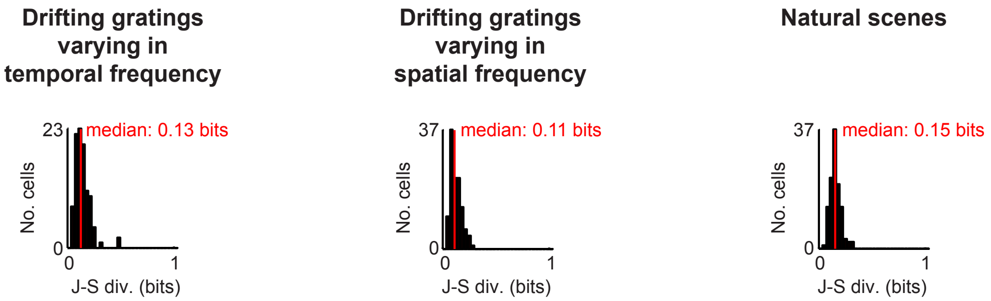

Figure S5. The posterior stimulus distributions of the model cells closely matched those of their real cell counterparts, as measured using the Jensen-Shannon (J-S) divergence. The figure shows histograms of the J-S α values for all cells in the data set (n=109, 120 and 113 cells for the three sets of stimuli, respectively). Briefly, as described in Methods, the α value is the median of the row-by-row divergences for a pair of matrices, where one matrix is produced by the model, and the other is produced by the real cell. The J-S divergences are calculated from the "plug-in" estimator, without regularization (because regularization is not required). The median α value across all stimulus sets is 0.14 (close to 0 on a 0 to 1 scale). Greater than 90% of the α values are less than 0.22. (Note that the J-S divergence is on a different scale from the K-L divergence, even though they're both in bits. For the J-S divergence, the scale is from 0 to 1 (the maximum J-S divergence is 1). For the K-L divergence, we used 0 to 4.9, since 4.9 bits is the stimulus entropy. For more detailed discussion of scale, see Fig. 4 legend.)
