# Supplementary material for: A Virtual Retina for Studying Population Coding
Source: PLoS One. 2013 Jan 14;8(1):e53363. doi: 10.1371/journal.pone.0053363 (PMC3544815; doi:10.1371/journal.pone.0053363)
Supplement: Figure S6 — Estimates of the Kullback-Leibler (K-L) divergence are not data-limited. This figure is a scattergram of the α values across all experiments, calculated as in the main text. The α values calculated from half the trials are plotted against the α values calculated from all the trials. As shown, the points lie close to the line of identity. (PDF) [file pone.0053363.s006.pdf]

Figure S6

Estimates of the Kullback-Leibler (K-L) divergence are not data-limited.

In Figure S6, we address the issue of data limitation in the context of our measure of the K-L divergence. Briefly, we determined the sensitivity of the calculated K-L divergence to the amount of data used. To do this, we repeated the comparison between the real and model cell posteriors (the calculation of Fig. 4B in the main text), but with only half of the trials, randomly chosen. As shown in Fig. S6, the conclusions still hold; the α values calculated using half the trials are similar to those obtained from all the trials, and are still very small. In quantitative terms, the difference between the analyses is 18%, with the α values tending to be smaller when all of the trials are used.

Note that the reason data limitation isn't an issue is that we're using posterior stimulus distributions, rather than maximum a posteriori (MAP). That is, for each trial, we obtain the probability that each stimulus occurred, given the response, and we use this full distribution, rather than just its peak value. So we retain more information from each trial than would have been the case had we used confusion matrices (which use just the peak value). So, even though the number of trials is close to the number of stimuli (25 trials, 30 stimuli), we don't become data limited, as the figure (Fig. S6) shows.

In sum, Fig. S6 shows that very similar results are obtained when only half the trials were used. Thus, the conclusion that there is only a small difference between the two distributions as measured by K-L divergence is robust. In addition, the conclusion is supported by two other measures, the J-S divergence (Fig. S5) and the MSE (Fig. 4), which also show that the difference between the two distributions is very small.

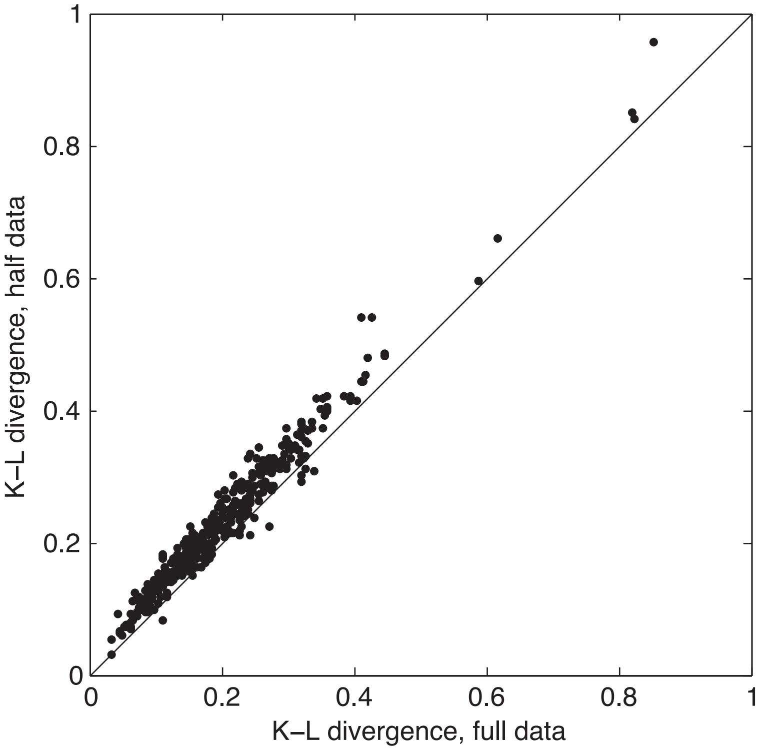

Figure S6. Estimates of the Kullback-Leibler (K-L) divergence are not data-limited. This figure is a scattergram of the α values across all experiments, calculated as in the main text. The α values calculated from half the trials are plotted against the α values calculated from all the trials. As shown, the points lie close to the line of identity.
