# Supplementary material for: A Virtual Retina for Studying Population Coding
Source: PLoS One. 2013 Jan 14;8(1):e53363. doi: 10.1371/journal.pone.0053363 (PMC3544815; doi:10.1371/journal.pone.0053363)
Supplement: Figure S7 — Raster plots for all cells viewing the stimulus set consisting of drifting gratings that varied in temporal frequency ( n = 109 cells). The stimulus is a continuous stream of drifting gratings with uniform gray fields interleaved (grating stimuli are 1 s, gray fields are 0.33 s). 5 s of a 41 s stimulus is shown (repeated 50 times). The vertical axis indicates the trials; 0 to 50 trials are shown for the real cell, followed by 0 to 50 trials for the model cell, following the layout in Figure 8 in the main text. The order of the rasters corresponds to the order of the posteriors in Figure S1. (PDF) [file pone.0053363.s007.pdf]

Figure S7

Raster plots for all cells viewing the stimulus set consisting of drifting gratings that varied in temporal frequency (n=109 cells). The stimulus is a continuous stream of drifting gratings with uniform gray fields interleaved (grating stimuli are 1 s, gray fields are 0.33 s). 5 s of a 41 s stimulus is shown (repeated 50 times). The vertical axis indicates the trials; 0 to 50 trials are shown for the real cell, followed by 0 to 50 trials for the model cell, following the layout in Figure 8 in the main text. The order of the rasters corresponds to the order of the posteriors in Figure S1.

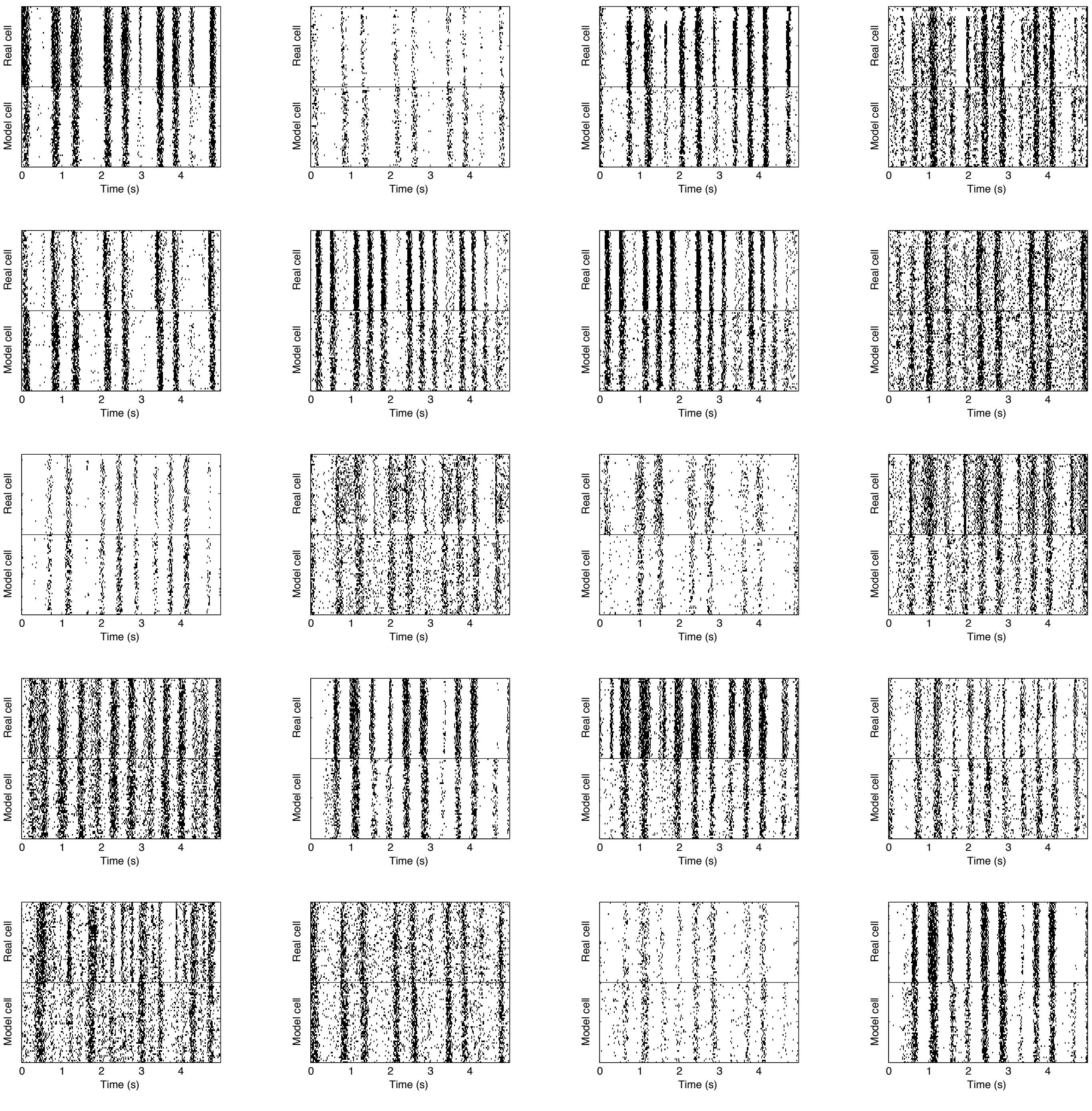

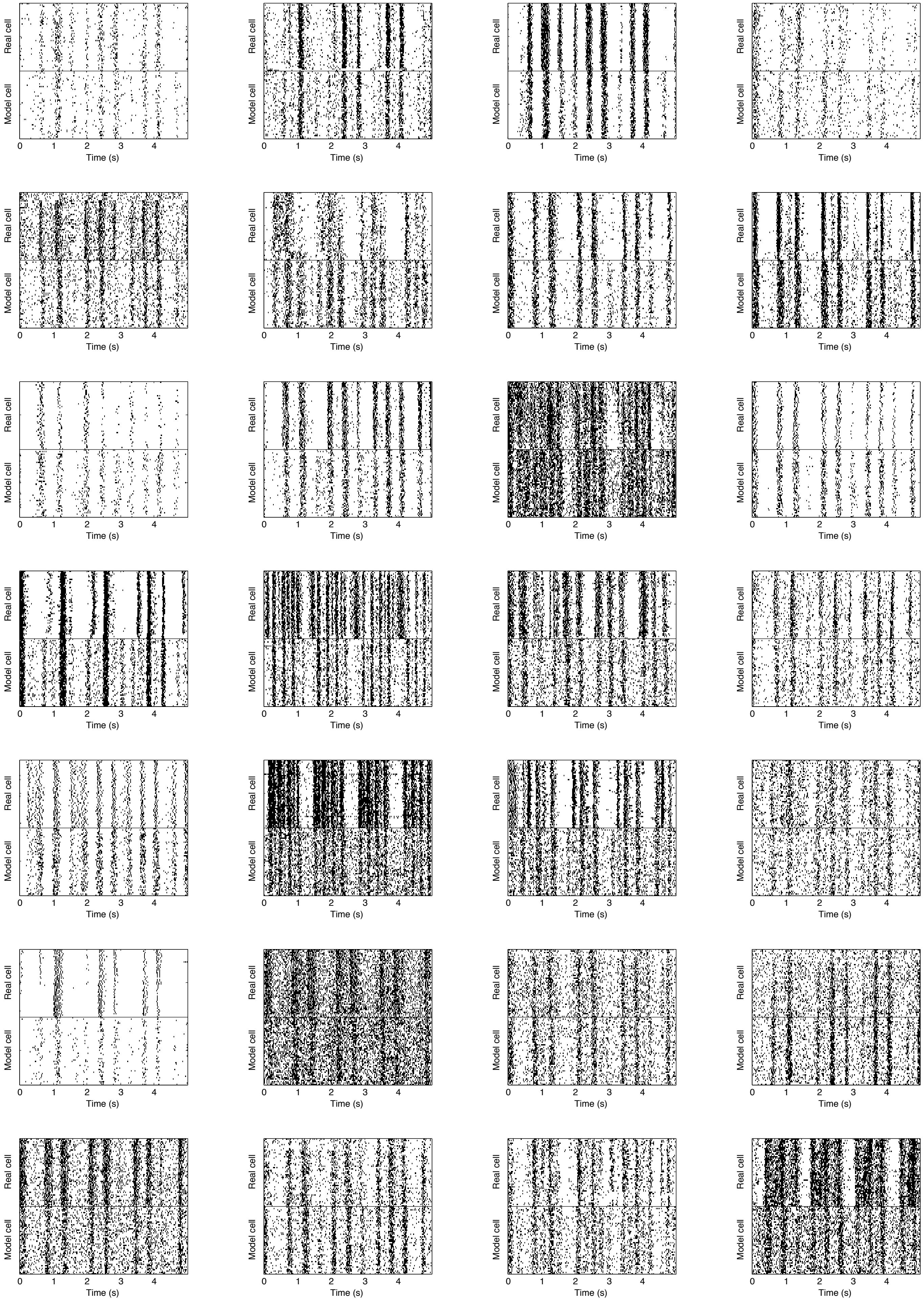
Real cell
Model cell
0 1 2 3 4
Time (s)

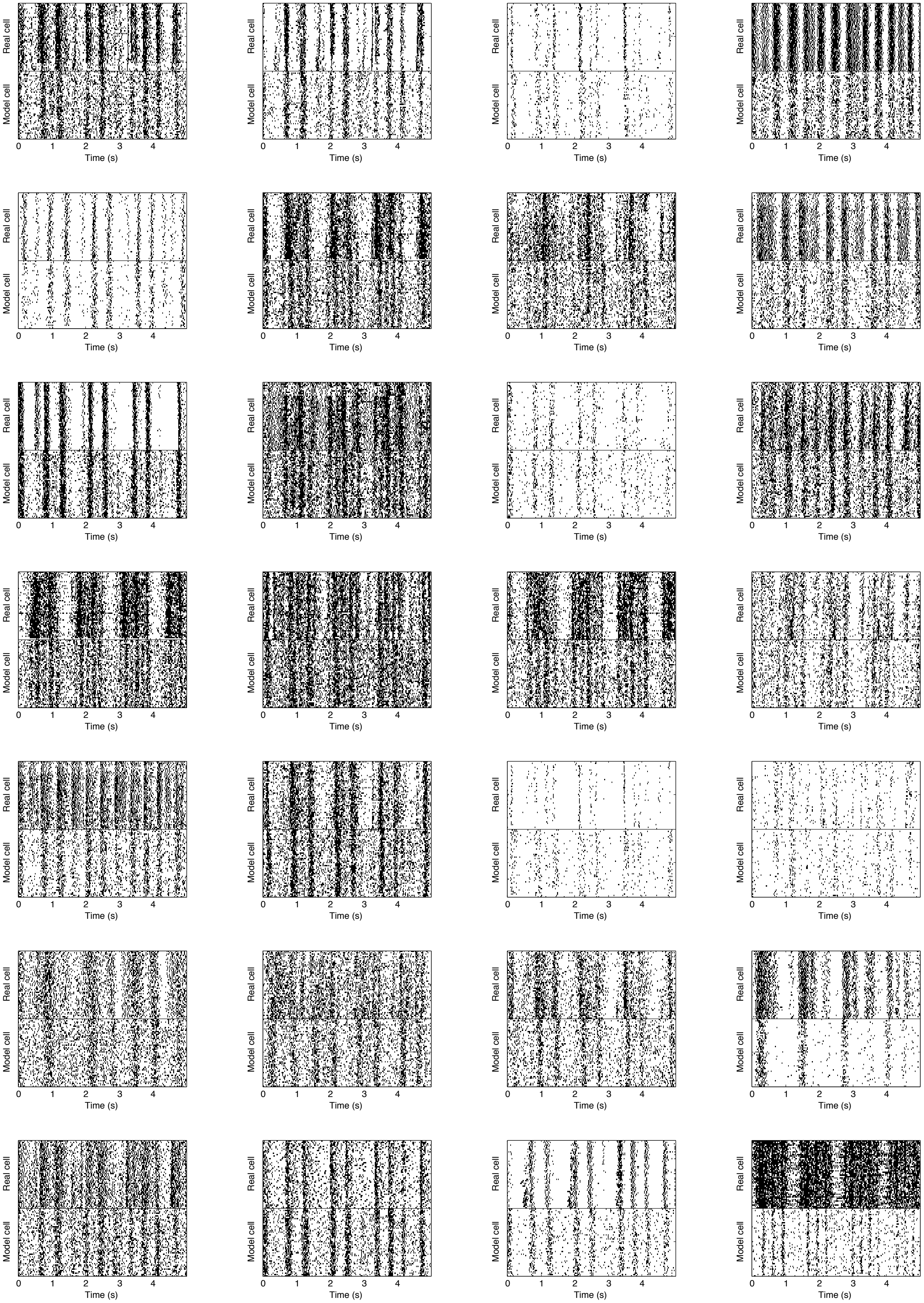

Real cell
Model cell
0
1
2
3
4
Time (s)

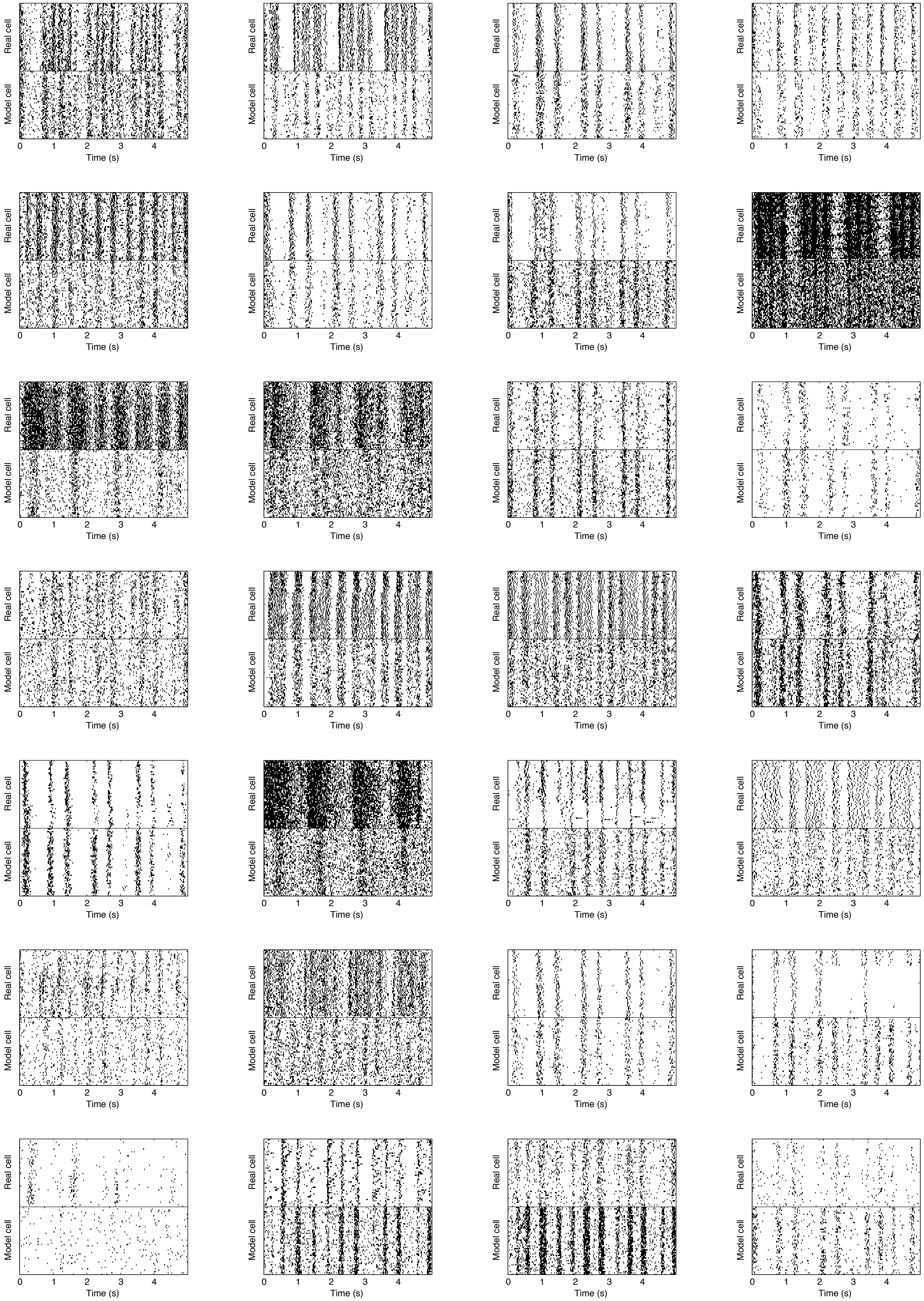
Real cell
Model cell
0 1 2 3 4
Time (s)

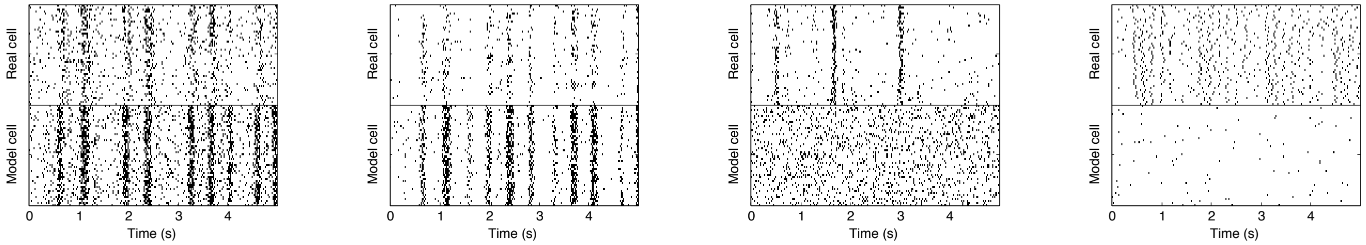

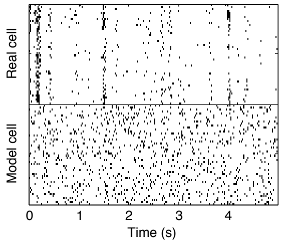

In 3 out of a total of 342 cells in the data set, the fitting procedure produced no model (as shown in the last three plots here); this is most likely because of experimental noise or lack of time for convergence. These cells are included for completeness: all cells with light driven responses were included in the dataset. As expected, these three cells show uniform average posteriors (shown in Fig. S1), and their MSE values are consistent with a uniform posterior, ~0.5 (see Methods).
