# Supplementary material for: A Virtual Retina for Studying Population Coding
Source: PLoS One. 2013 Jan 14;8(1):e53363. doi: 10.1371/journal.pone.0053363 (PMC3544815; doi:10.1371/journal.pone.0053363)
Supplement: Figure S8 — Raster plots for all cells viewing the stimulus set consisting of drifting gratings that varied in spatial frequency ( n = 120 cells). The stimulus is a continuous stream of drifting gratings with uniform gray fields interleaved (grating stimuli are 1 s, gray fields are 0.33 s). 5 s of a 41 s stimulus is shown (repeated 50 times). The vertical axis indicates the trials; 0 to 50 trials are shown for the real cell, followed by 0 to 50 trials for the model cell, following the layout in Figure 8 in the main text. The order of the rasters corresponds to the order of the posteriors in Figure S2. (PDF) [file pone.0053363.s008.pdf]

Figure S8

Raster plots for all cells viewing the stimulus set consisting of drifting gratings that varied in spatial frequency (n=120 cells). The stimulus is a continuous stream of drifting gratings with uniform gray fields interleaved (grating stimuli are 1 s, gray fields are 0.33 s). 5 s of a 41 s stimulus is shown (repeated 50 times). The vertical axis indicates the trials; 0 to 50 trials are shown for the real cell, followed by 0 to 50 trials for the model cell, following the layout in Figure 8 in the main text. The order of the rasters corresponds to the order of the posteriors in Figure S2.

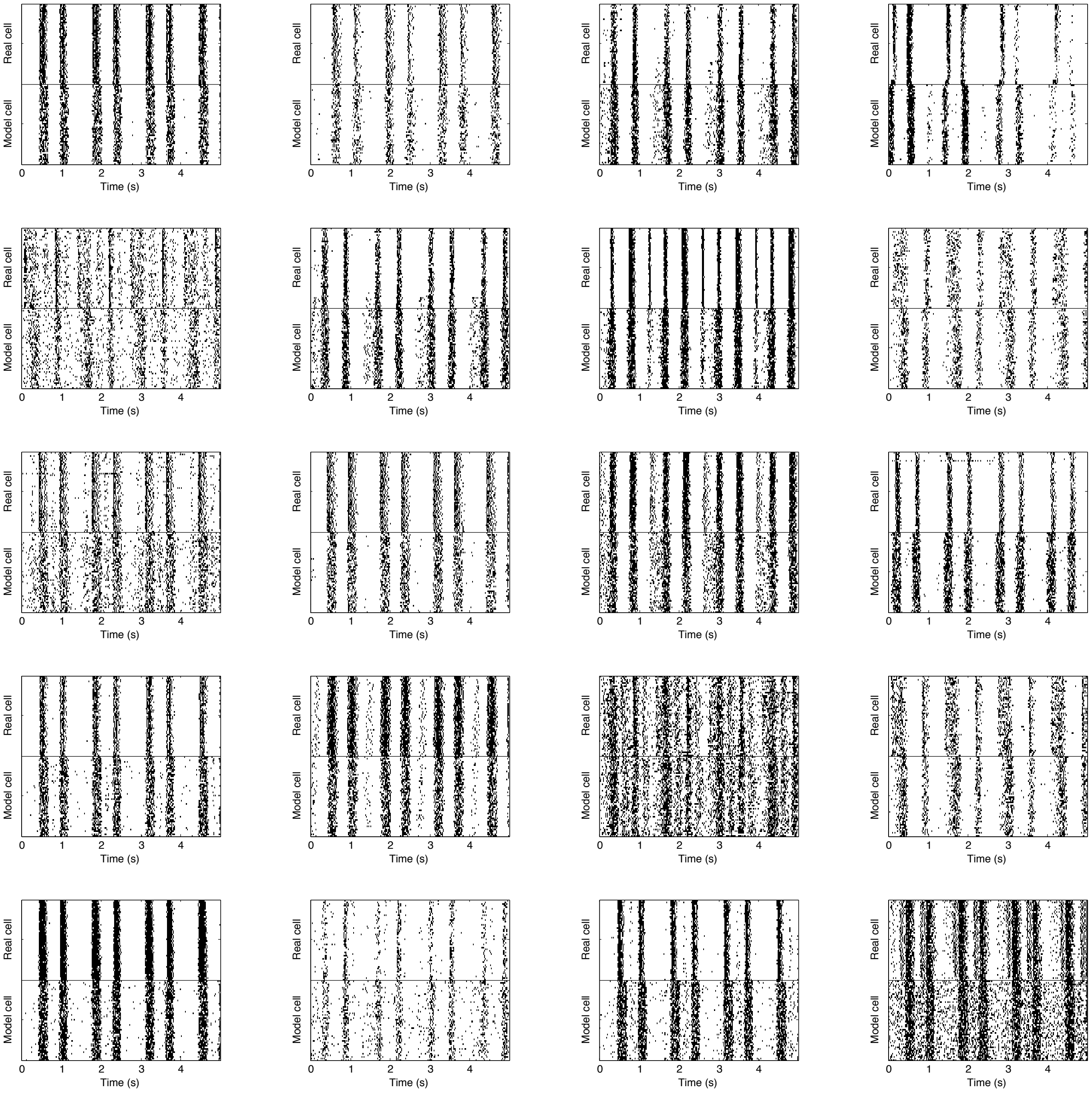

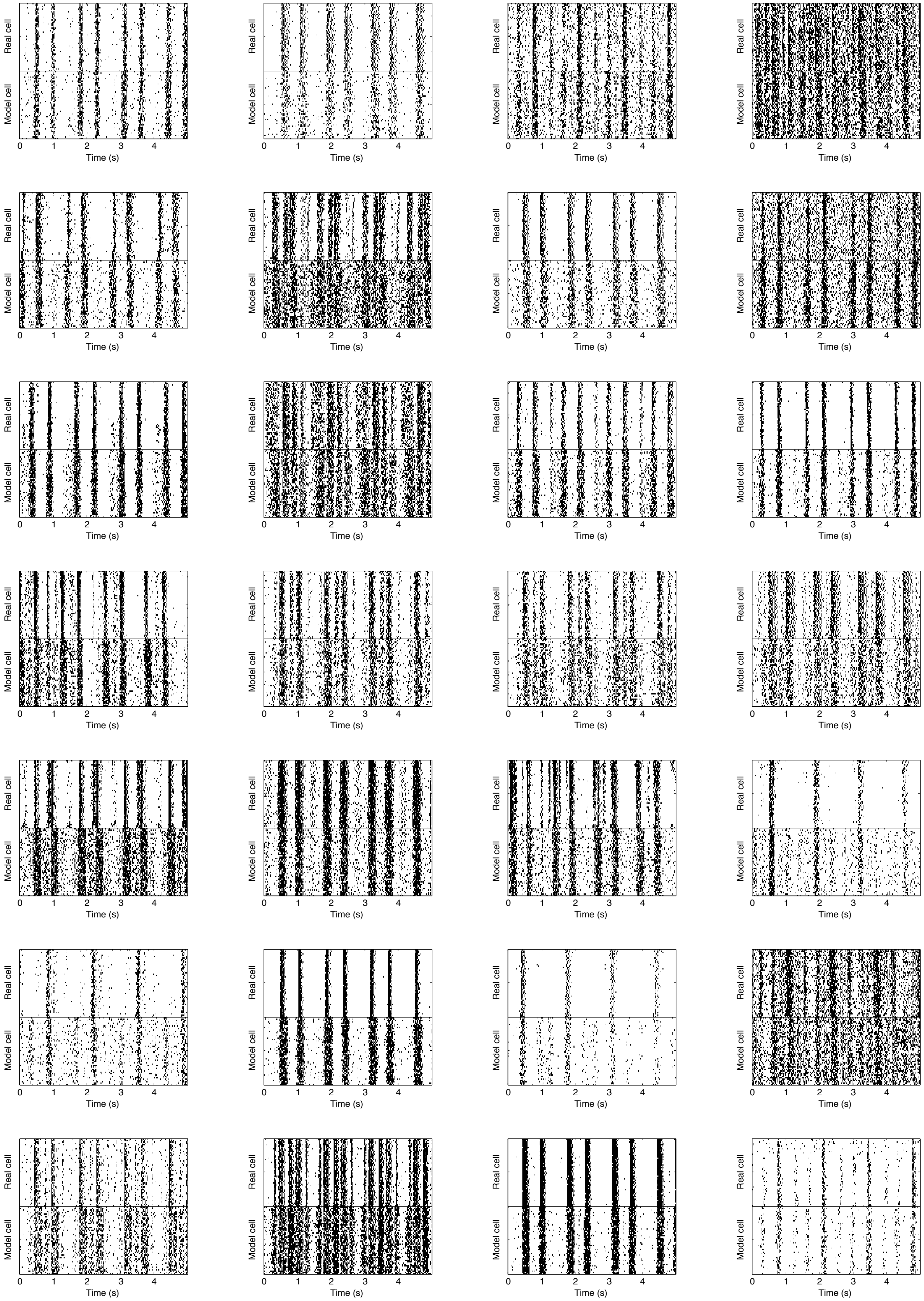
Real cell
Model cell
0
1
2
3
4
Time (s)

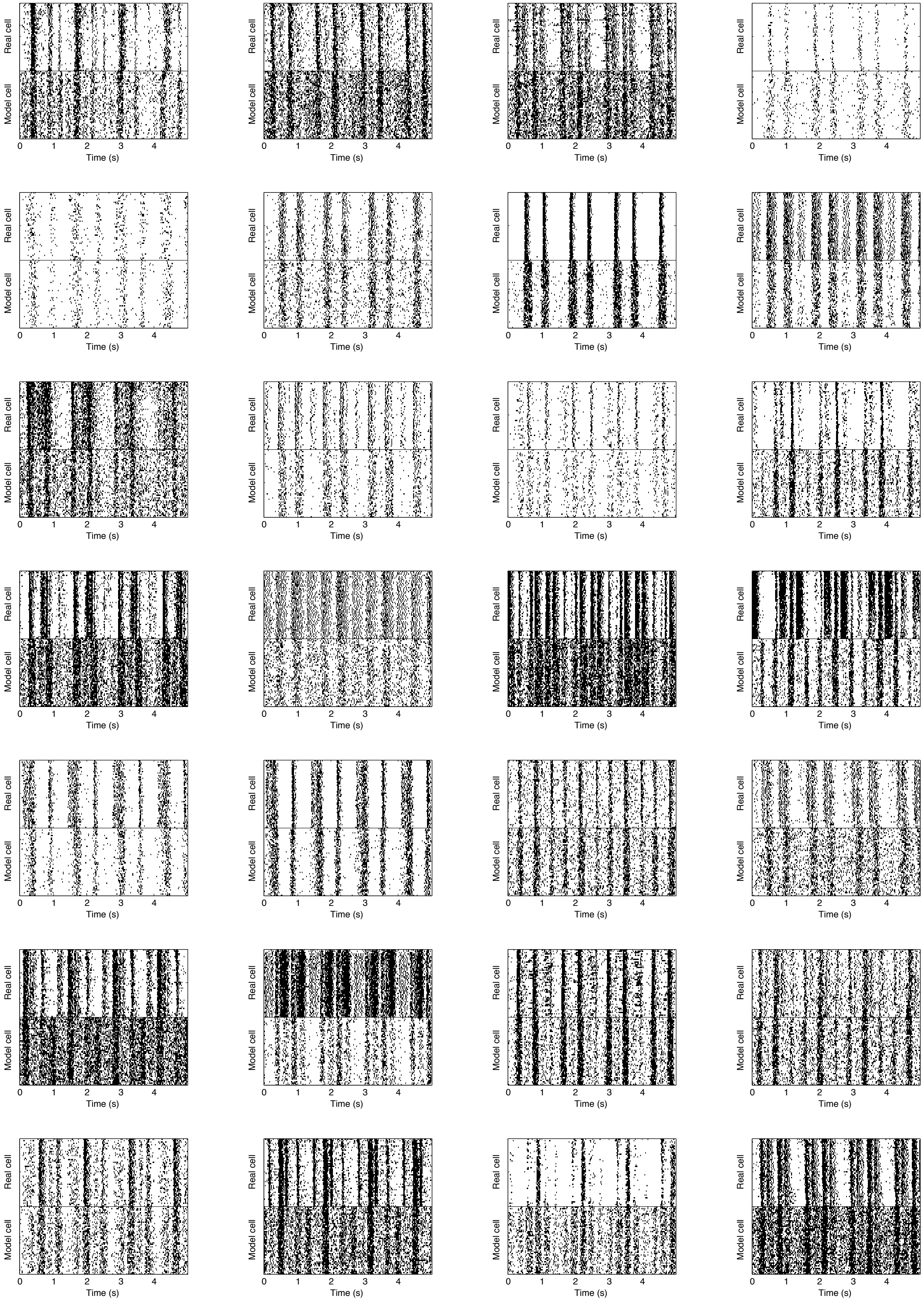
Real cell
Model cell
Time (s)
0 1 2 3 4

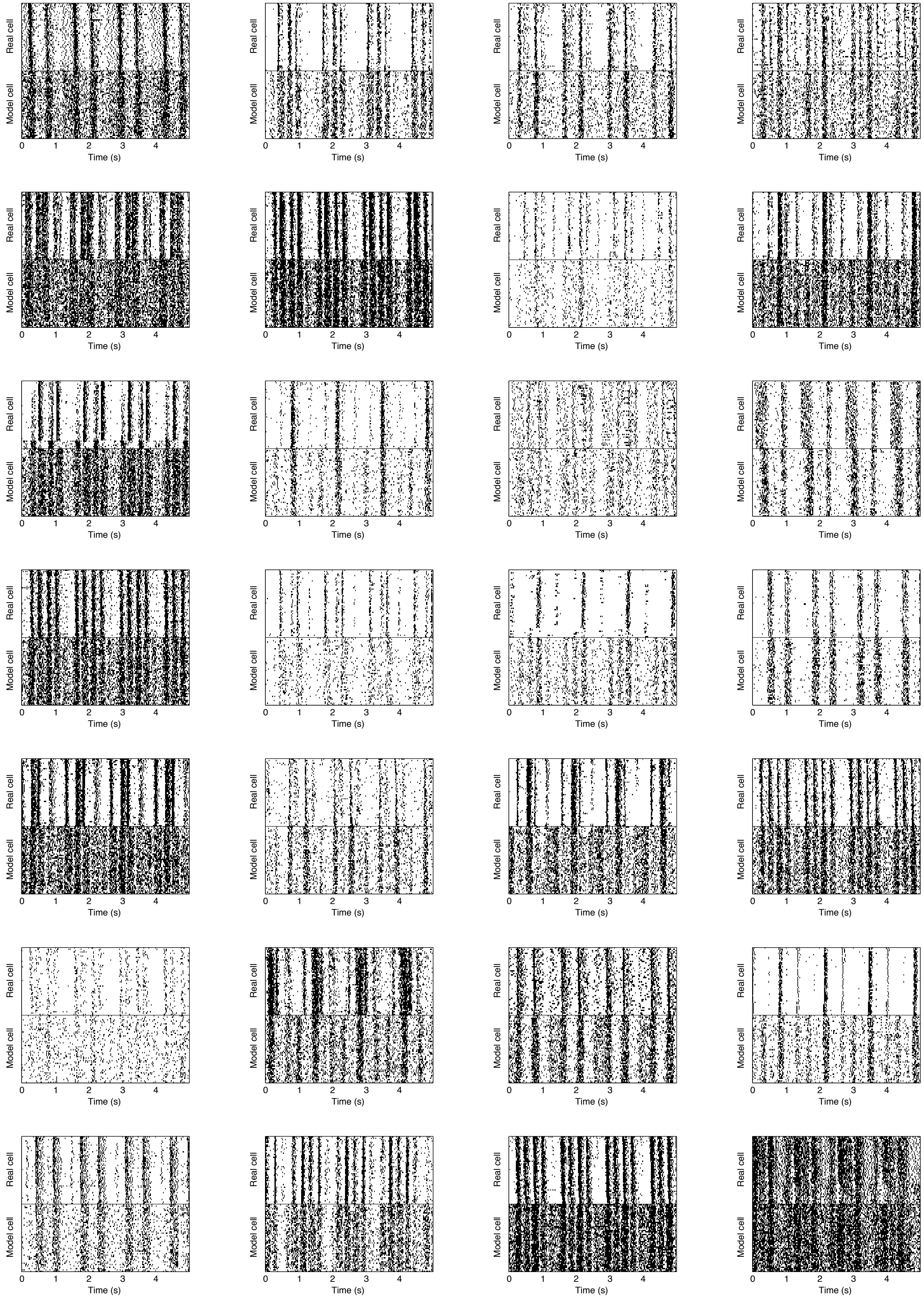

Real cell
Model cell
0 1 2 3 4
Time (s)

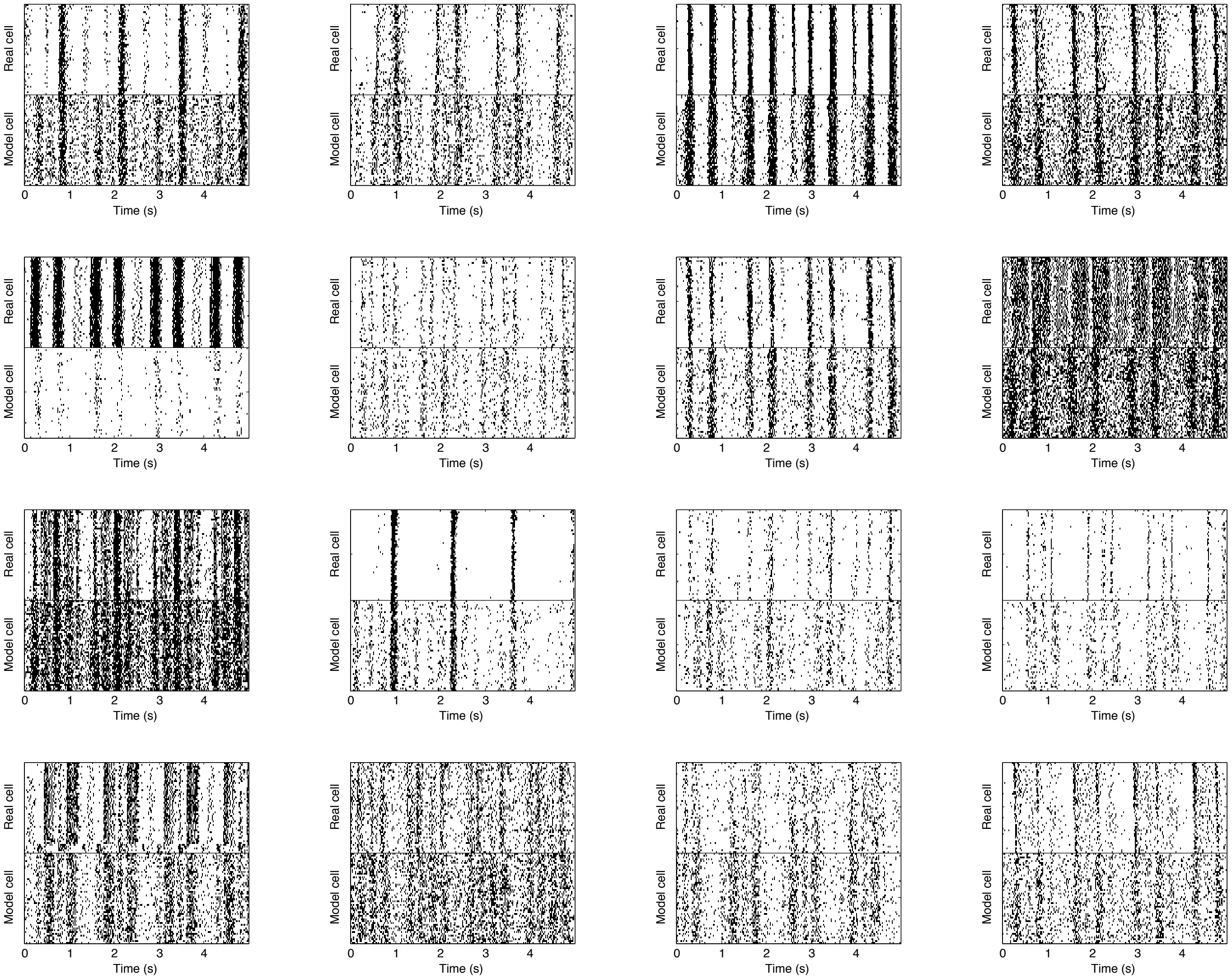
Real cell
Model cell
0
1
2
3
4
Time (s)
