# Supplementary material for: A Virtual Retina for Studying Population Coding
Source: PLoS One. 2013 Jan 14;8(1):e53363. doi: 10.1371/journal.pone.0053363 (PMC3544815; doi:10.1371/journal.pone.0053363)
Supplement: Figure S9 — Raster plots for all cells viewing the stimulus set consisting of natural scenes ( n = 113 cells). The stimulus is a continuous stream of natural movies with uniform gray fields interleaved (natural movies are 1 s long, gray fields are 0.33 s). Note that each cell is viewing a different location in the movie: this is the case for the cells in Figs. S7 and S8 as well, but it is most obvious here in the natural scene rasters, since the movie is not a periodic stimulus. 5 s of a 41 s stimulus is shown (repeated 50 times). The vertical axis indicates the trials; 0 to 50 trials are shown for the real cell, followed by 0 to 50 trials for the model cell, following the layout in Figure 8 in the main text. The order of the rasters corresponds to the order of the posteriors in Figure S3. (PDF) [file pone.0053363.s009.pdf]

Figure S9

Raster plots for all cells viewing the stimulus set consisting of natural scenes (n=113 cells). The stimulus is a continuous stream of natural movies with uniform gray fields interleaved (natural movies are 1 s long, gray fields are 0.33 s). Note that each cell is viewing a different location in the movie: this is the case for the cells in Figs. S7 and S8 as well, but it is most obvious here in the natural scene rasters, since the movie is not a periodic stimulus. 5 s of a 41 s stimulus is shown (repeated 50 times). The vertical axis indicates the trials; 0 to 50 trials are shown for the real cell, followed by 0 to 50 trials for the model cell, following the layout in Figure 8 in the main text. The order of the rasters corresponds to the order of the posteriors in Figure S3.

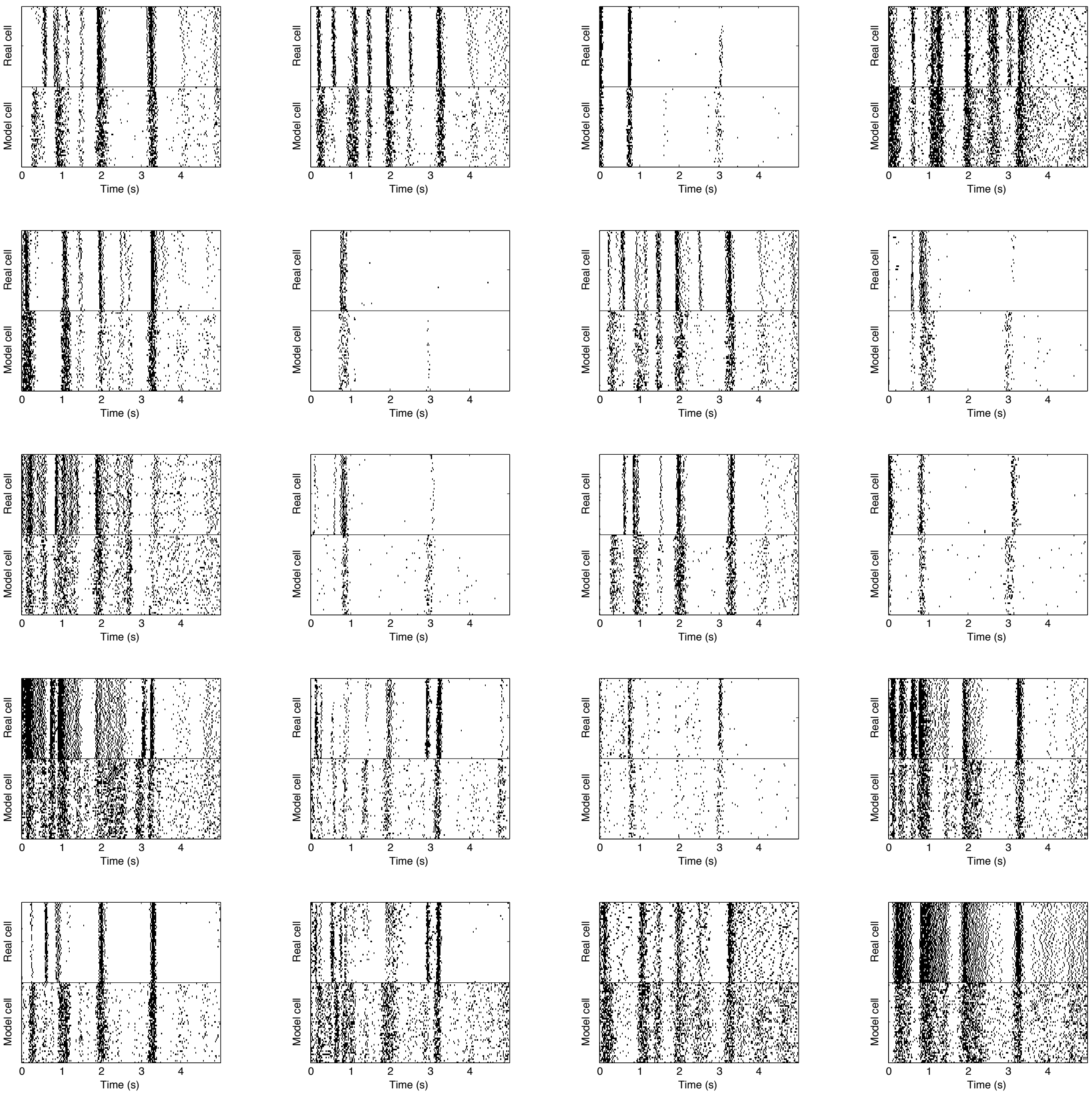

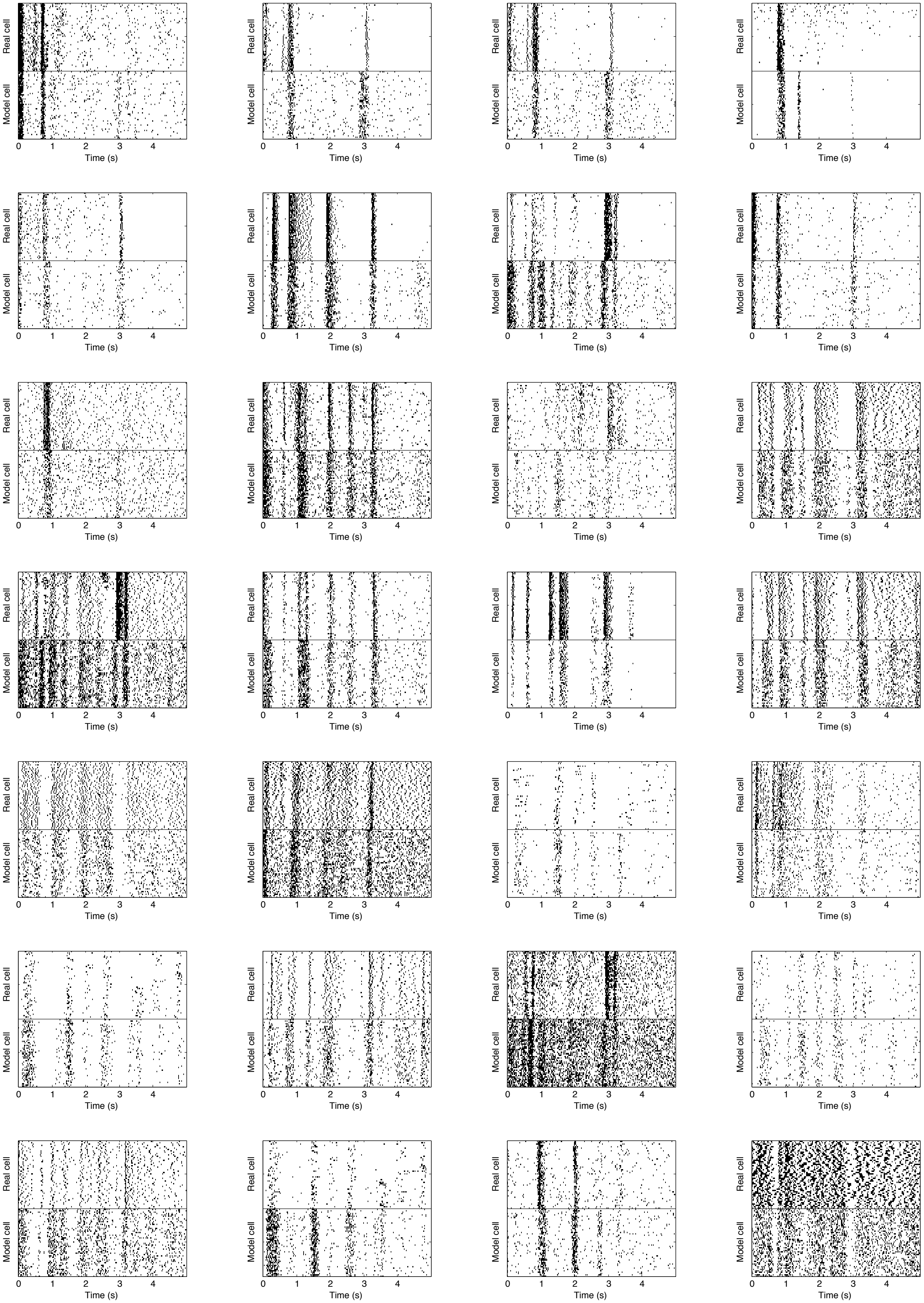
Real cell
Model cell
0 1 2 3 4
Time (s)

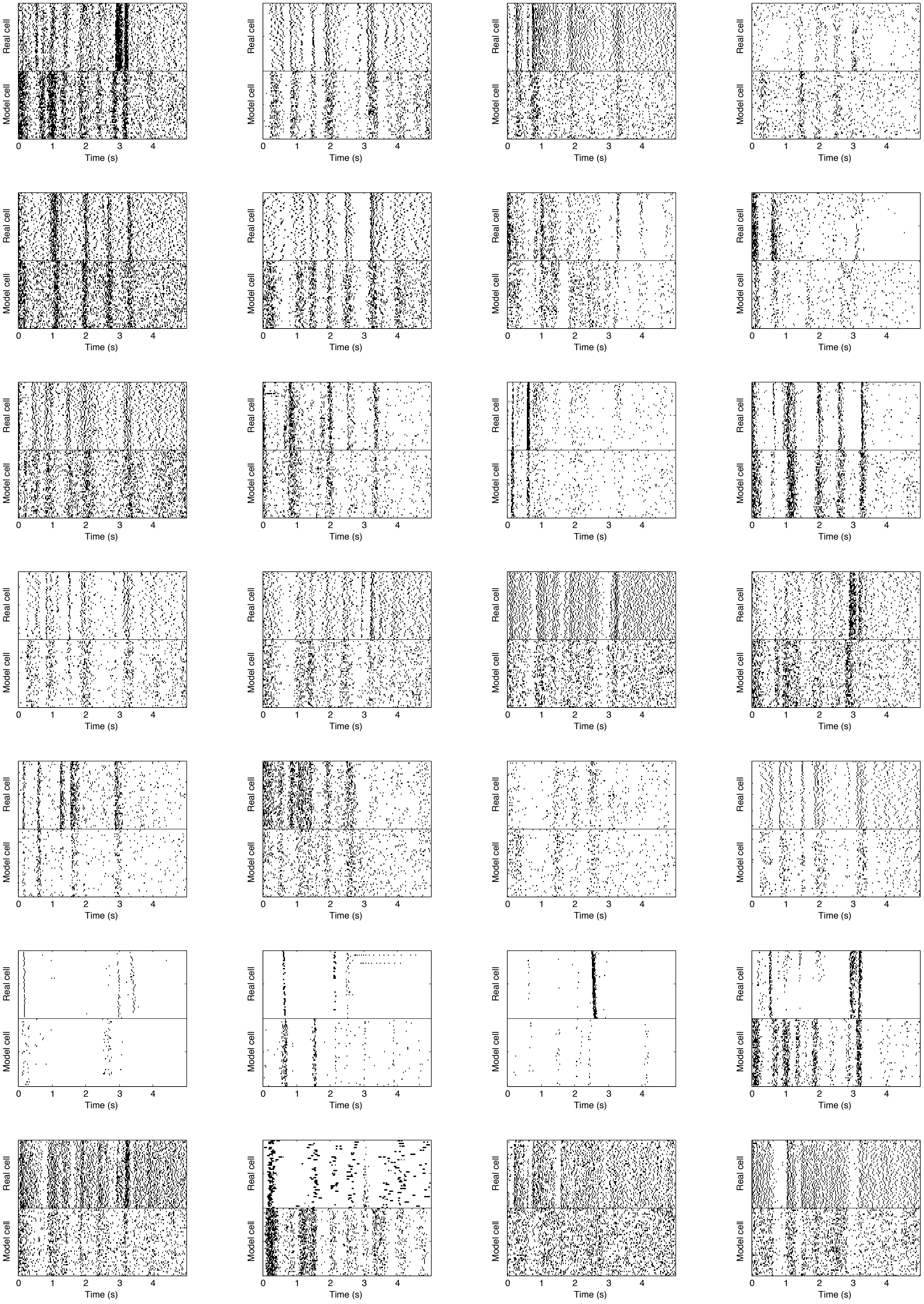
Real cell
Model cell
Time (s)
0 1 2 3 4

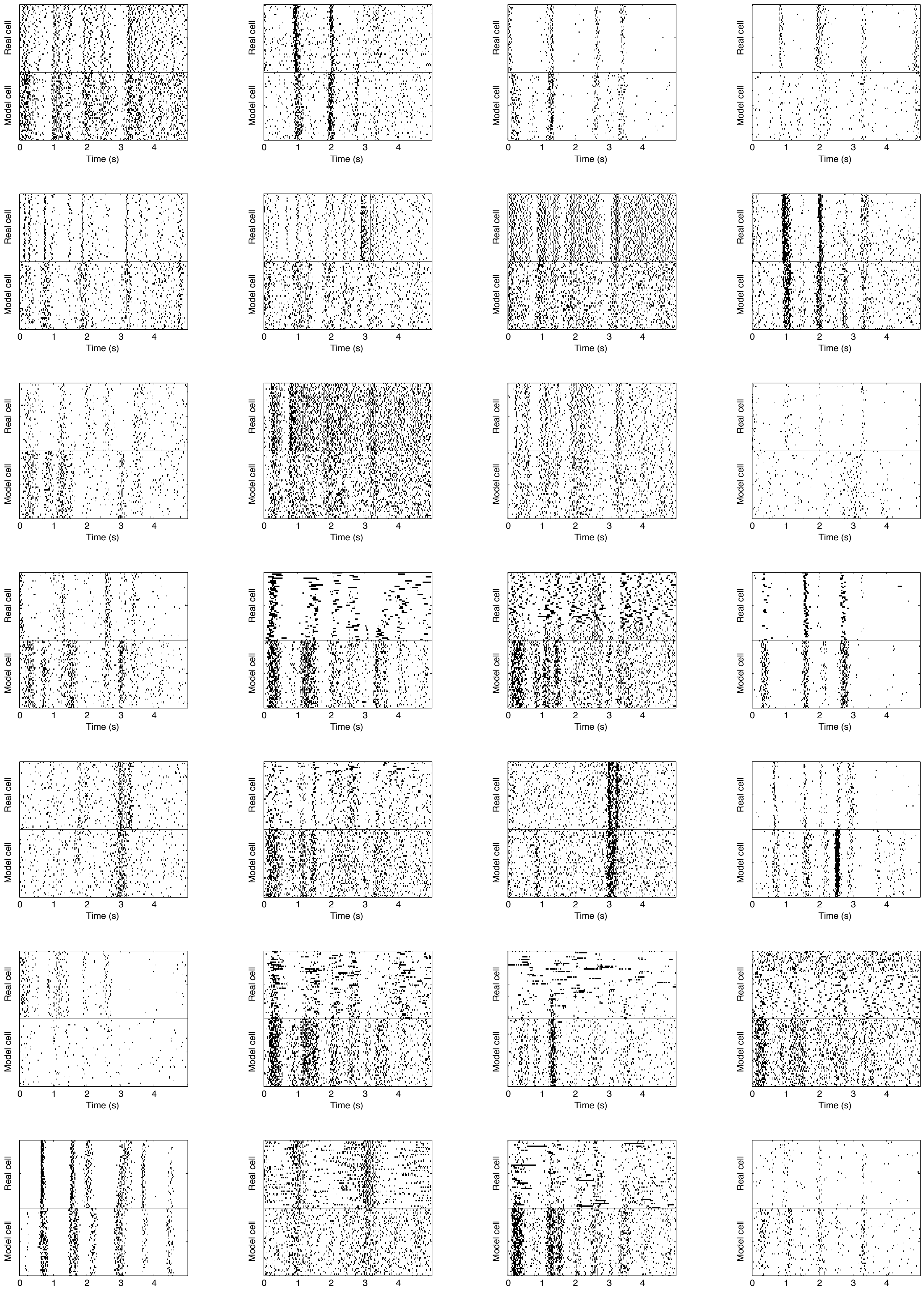

Real cell
Model cell
0
1
2
3
4
Time (s)

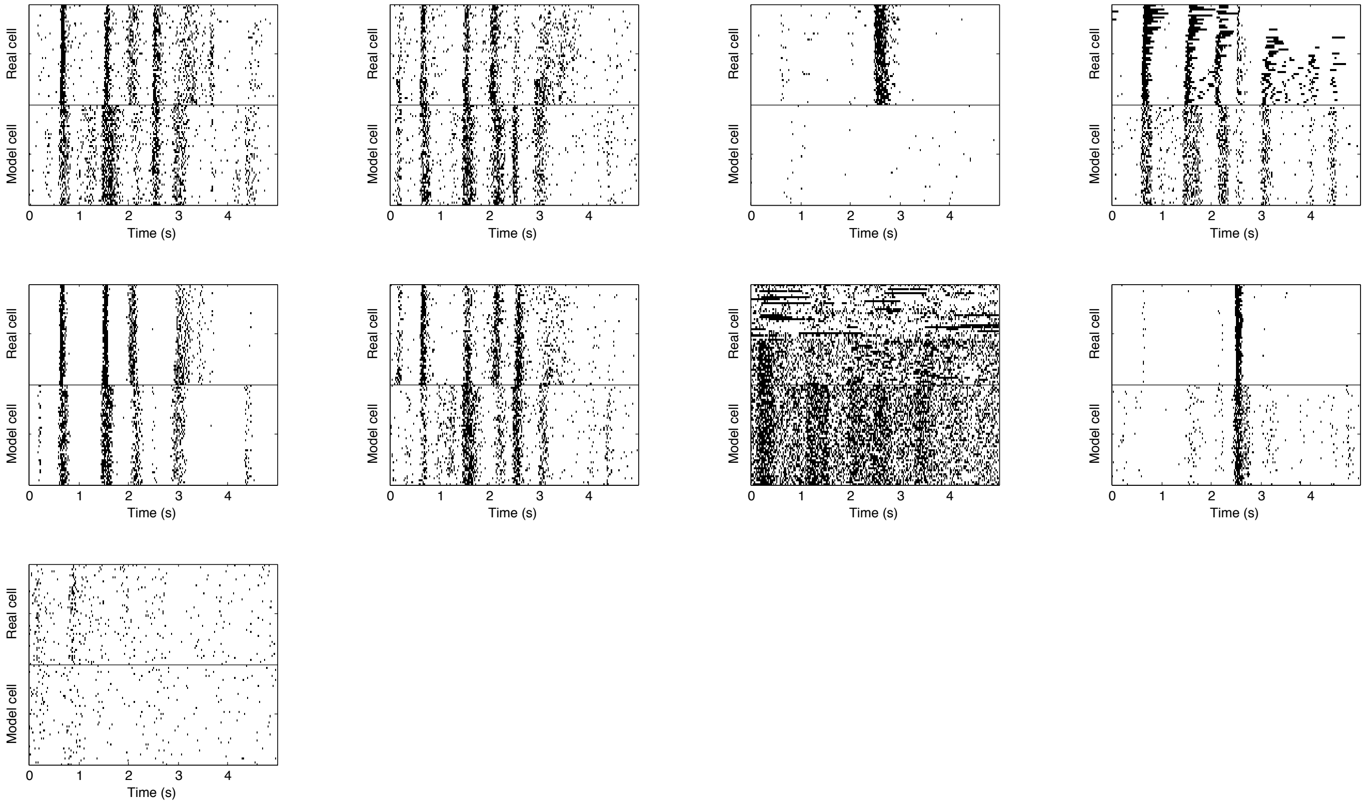
Real cell
Model cell
0
1
2
3
4
Time (s)
